# Skin Temperature Measurement Using Contact Thermometry: A Systematic Review of Setup Variables and Their Effects on Measured Values

**DOI:** 10.3389/fphys.2018.00029

**Published:** 2018-01-30

**Authors:** Braid A. MacRae, Simon Annaheim, Christina M. Spengler, René M. Rossi

**Affiliations:** ^1^Empa, Swiss Federal Laboratories for Materials Science and Technology, Laboratory for Biomimetic Membranes and Textiles, St. Gallen, Switzerland; ^2^Exercise Physiology Lab, Institute of Human Movement Sciences and Sport, Department of Health Sciences and Technology, ETH Zurich, Zurich, Switzerland; ^3^Zurich Center for Integrative Human Physiology, University of Zurich, Zurich, Switzerland

**Keywords:** skin temperature, thermometry, measurement error, comparability, agreement, validity, bias

## Abstract

**Background:** Skin temperature (*T*_skin_) is commonly measured using *T*_skin_ sensors affixed directly to the skin surface, although the influence of setup variables on the measured outcome requires clarification.

**Objectives:** The two distinct objectives of this systematic review were (1) to examine measurements from contact *T*_skin_ sensors considering equilibrium temperature and temperature disturbance, sensor attachments, pressure, environmental temperature, and sensor type, and (2) to characterise the contact *T*_skin_ sensors used, conditions of use, and subsequent reporting in studies investigating sports, exercise, and other physical activity.

**Data sources and study selection:** For the measurement comparison objective, Ovid Medline and Scopus were used (1960 to July 2016) and studies comparing contact *T*_skin_ sensor measurements *in vivo* or using appropriate physical models were included. For the survey of use, Ovid Medline was used (2011 to July 2016) and studies using contact temperature sensors for the measurement of human *T*_skin_ *in vivo* during sport, exercise, and other physical activity were included.

**Study appraisal and synthesis methods:** For measurement comparisons, assessments of risk of bias were made according to an adapted version of the Cochrane Collaboration's risk of bias tool. Comparisons of temperature measurements were expressed, where possible, as mean difference and 95% limits of agreement (LoA). Meta-analyses were not performed due to the lack of a common reference condition. For the survey of use, extracted information was summarised in text and tabular form.

**Results:** For measurement comparisons, 21 studies were included. Results from these studies indicated minor (<0.5°C) to practically meaningful (>0.5°C) measurement bias within the subgroups of attachment type, applied pressure, environmental conditions, and sensor type. The 95% LoA were often within 1.0°C for *in vivo* studies and 0.5°C for physical models. For the survey of use, 172 studies were included. Details about *T*_skin_ sensor setup were often poorly reported and, from those reporting setup information, it was evident that setups widely varied in terms of type of sensors, attachments, and locations used.

**Conclusions:** Setup variables and conditions of use can influence the measured temperature from contact *T*_skin_ sensors and thus key setup variables need to be appropriately considered and consistently reported.

## Introduction

Heat exchanges at the skin surface can both contribute to and challenge thermal homeostasis. Accordingly, quantification of skin temperature (*T*_skin_) is important in many research and applied settings and using sensors affixed directly to the skin surface is common for this purpose. While measuring *T*_skin_ in this way is simple in terms of access to the measurement site, an inherent challenge is that accurate measurements of surface temperature are difficult to accomplish (Hardy, 1934b; Malone, 1963; Boetcher et al., 2009). Understanding measurement limitations benefits users by supporting decisions during sensor selection and setup, use, and interpretation of resultant data, and can assist in the development of future sensor systems.

Human *T*_skin_ data are used widely, including for evaluating thermal strain (Moran et al., 1999; International Organization for Standardization, 2004; McLellan et al., 2013), estimating mean body temperature (Burton, 1935; Colin et al., 1971; Lenhardt and Sessler, 2006) and body heat content and storage by thermometry (Tucker et al., 2006) (although with limitation compared to whole body calorimetry; Jay et al., 2007, 2010), and validating psychophysical and thermophysiological models (Zhang et al., 2010; Martinez et al., 2016). Further, *T*_skin_ is used for understanding the mechanisms of acute thermoregulatory and cardiovascular responses (Rowell et al., 1969; Nadel et al., 1971; Libert et al., 1978; Cotter and Taylor, 2005) and adaptation to thermal stress (Regan et al., 1996), understanding how perturbations of body temperature, and strategies to mitigate or recover from such perturbations, influence aspects of sports, and occupational performance (Daanen, 2009; Sawka et al., 2011, 2012; Schlader et al., 2011a; Caldwell et al., 2012; Levels et al., 2012; Ross et al., 2013; Lee et al., 2015; Stevens et al., 2017), and understanding effects of the ambient environment (Gagge et al., 1967; Galloway and Maughan, 1997) or body coverings (Gagge et al., 1938; Nielsen et al., 1985; White and Hodous, 1987; Ruckman et al., 1999; Rossi, 2003; Barwood et al., 2016). With growing interest in wearable technology and body monitoring, the prospects for applied uses of *T*_skin_ data are also expanding and include monitoring of individuals during sport and exercise or working under extreme conditions.

Contact thermometry consists of a temperature sensor positioned in direct contact with the skin surface and, therefore, relies on conductive heat exchanges between skin and sensor. Contact thermometry is popular for measuring *T*_skin_ in both research and applied settings with reasons including commercial sensor availability and relative low cost, small sensor size and potential robustness, ease of measurement continuity within a measurement period, the ability to position sensors at a selection of body sites, and that measurements can be made at sites under coverings like clothing and protective equipment. These sensors, however, can differ in terms of the underlying measurement principle (e.g., electrical resistance for thermistors, Seebeck effect for thermocouples), sensor size and shape, constituent materials, and resultant thermal properties. The method of attachment to the skin also varies, with tape or other adhesive common in research settings (Buono and Ulrich, 1998; Psikuta et al., 2014). With the variety of contact *T*_skin_ sensors and attachments commonly used, it is necessary to recognise if equivalent measurements can be expected irrespective of the sensor type and setup variables.

Issues regarding validity of data from contact *T*_skin_ sensors also require consideration. That is, do these sensors actually measure what they are supposed to be measuring? Due to inherent skin contact and coverage at the measured sites, a disadvantage of contact thermometry is that the sensors and their attachments modify the immediate environment for the underlying skin. The effects of such a modification may manifest as a disturbance of the temperature that would otherwise exist in the undisturbed case (Henriques, 1947; Childs et al., 2000; Boetcher et al., 2009; Taylor et al., 2014). Further, the *T*_skin_ is not measured directly: the temperature measured is that of the sensor itself (Malone, 1963; Childs, 2001) and, therefore, it is important to consider whether the sensor temperature is at equilibrium with the underlying skin temperature. Non-contact techniques utilising electromagnetic radiation emitted from the skin surface—therefore, able to measure temperature from a distance—can overcome certain difficulties associated with sensor contact and coverage (Hardy, 1934a,b; Hardy and Soderstrom, 1937), but have difficulties of their own, including limited practicality during movement or field use, influences of changes in the emissivity of the skin surface (Bernard et al., 2013), and unclear validity of common commercial devices, particularly during exercise (Bach et al., 2015b). Thus, no method is without limitation and, while imperfect, the general practicality of contact thermometry likely means it will remain a desirable method for quantifying *T*_skin_ in the foreseeable future.

To elucidate relevant considerations for the measurement of *T*_skin_ using contact thermometry, we systematically reviewed the literature in accordance with two distinct objectives. The primary objective, objective 1, was to examine measurements from contact *T*_skin_ sensors considering sensor-surface equilibrium temperature and surface temperature disturbance, and considering whether setup variables (sensor type, attachments, applied pressure, or environmental conditions) meaningfully influence the measured temperature. The secondary objective, objective 2, was to survey and characterise the contact *T*_skin_ sensors used, conditions of use, and subsequent reporting of *T*_skin_ in published scientific studies investigating sport, exercise, and other physical activity.

## Methods

A protocol was developed before the formal searches were performed, specifying the literature searches, screening, inclusion, and data synthesis. The review was conducted in general accordance with PRISMA guidelines (Moher et al., 2009).

### Search strategy

The search strategies (including full lists of search terms) used for both objectives are given in Supplementary Material Tables 1, 2. In brief, terms including and relating to skin, temperature, and thermometry were used for the measurement comparisons (objective 1), and terms including and relating to skin, temperature, sport, exercise, and physical activity were used for the survey of use (objective 2). Searches were performed 13.07.2016.

The electronic databases Ovid Medline and Scopus were used for objective 1. Reference lists of studies meeting inclusion criteria were searched for other relevant articles and Google Scholar was used to identify any relevant articles citing included studies. The electronic database Ovid Medline was used for objective 2. One database was considered appropriate because, given the purpose of this objective and the breadth of journals included in Medline, the practicality was considered to outweigh the risk of bias for publication sampling. No further articles were added for objective 2 from other search means. No language restrictions were imposed for the searches. Studies published in the grey literature or in truncated form (e.g., abstracts only, posters) were not considered.

### Study eligibility criteria

#### Measurement comparisons

We sought temperature data from contact temperature sensors. Studies using “conventional” contact temperature sensors—those that can be affixed to the skin surface to measure *T*_skin_—were included; other contact sensors (including those temporarily held against the skin) were included only in cases where they provided insight into setup considerations for affixable sensors. Measurements from liquid-crystal thermometers or other colour-change materials and measurements from non-contact methods were excluded. Studies were included if human or other animal *T*_skin_ was measured *in vivo*; surface temperature (*T*_surface_) measurements of a physical model were also included if the model was used in the context of understanding human *T*_skin_ measurement and if the temperatures investigated were physiologically relevant for humans. Included studies were required to report the measurement of a suitable comparison temperature, typically another *T*_skin_ or *T*_surface_ (the comparator determined to what outcome subgroup the data belonged; see data synthesis below). Comparisons performed simply for the purpose of validating or correcting a prototype sensor system were excluded (e.g., Chen et al., 2015). Comparisons with non-contact methods or numerical models, while useful in their own right, were beyond the scope of this article. (The comparison of contact and infra-red devices for measuring *T*_skin_ has been reviewed elsewhere; Bach et al., 2015b.) Studies investigating *T*_skin_ or *T*_surface_ during clinical or rehabilitation treatments/conditions were excluded (e.g., cryotherapy, ultrasound therapy, radiotherapy, other hyperthermia therapy). Studies published from 1960 (until 13.07.2016) were considered for inclusion, which was a modification from the unrestricted date range specified in the protocol (see Supplementary Material Appendix 1).

#### Survey of use

We sought general information about *T*_skin_ sensors and their use in published research (e.g., sensor type, attachments, conditions of sensor use, and use of the *T*_skin_ data). Studies included were those that used contact temperature sensors for the measurement of human *T*_skin_ *in vivo* during experimental studies involving sport, exercise, and other physical activity (PA; hereafter for practicality, sport, exercise, occupational and other PA will be collectively termed PA). The sensors had to be affixed to the skin surface in some way and remain in place during the PA. While measurement of *T*_skin_ is relevant beyond situations involving PA (e.g., clinical use, passive heat stress, sleep studies, circadian rhythm studies), measurements in these contexts were beyond the scope of this review.

For inclusion, studies needed to report *T*_skin_ data recorded during or immediately following PA, or use that data to calculate another variable that was reported in the results. Studies were excluded if they involved only “trivial” PA, such as standing tasks. Studies in which it was clear the *T*_skin_ data had been previously published in article form and studies involving daily monitoring were excluded unless that monitoring was part of a specific occupational routine including an expected component of PA. For objective 2, only studies published in the years 2011–2016 (until 13.07.2016) were included to be reflective of current practice at the time of review.

### Study selection and data extraction

All records were screened against the eligibility criteria first by title and abstract then, for those remaining, by full text. For measurement comparisons, duplicates were removed and preliminary inclusion of studies was performed by one investigator (BM). Final inclusion was performed after a systematic cross-check (SA or CS); disagreements were resolved by discussion among the three investigators. For the survey of use, one investigator (BM) performed preliminary inclusion. Final inclusion was performed after a systematic cross-check (SA) and any disagreements were resolved by discussion with a third investigator (CS).

For measurement comparisons, one investigator (BM) extracted study information and temperature data, with a second investigator (SA) verifying the accuracy. Disagreements were solved by discussion including a third investigator (CS). Temperature data were typically extracted from text or tables; for eight studies it was necessary to extract data, at least in part, from figures (Flesch et al., 1976; Dollberg et al., 1994; Buono and Ulrich, 1998; van Marken Lichtenbelt et al., 2006; Deng and Liu, 2008; Youhui et al., 2010; McFarlin et al., 2015; Priego Quesada et al., 2015) and original data were available for two studies (Psikuta et al., 2014; Bach et al., 2015a). Data extracted from figures was done so using a computer-based extraction tool (http://arohatgi.info/WebPlotDigitizer/). For the survey of use, one investigator extracted study information (BM) and at least one other investigator was consulted to discuss uncertainties. Spreadsheets were produced and refined during piloting work with input from multiple investigators and common problems were discussed before formal data extraction. Further details about the extracted data information are given in Supplementary Material Appendix 2.

### Assessments of risk of bias

For measurement comparisons, assessments of risk of bias were made at the outcome subset level according to an adapted version of the Cochrane Collaboration's risk of bias tool (Higgins et al., 2011) (see Supplementary Material Table 3 for the specific criteria used). Seven sources of bias were assessed: four that are widely used (sequence generation, blinding of participants and personnel, incomplete outcome data, selective reporting) and three specific “other” sources of bias (consistency of test conditions, calibration/baseline comparability of sensors, and study support). Assessments were made independently by two investigators (BM, SA); a third investigator was available for mediation but was not required. Sources of bias were judged as low, unclear, or high risk of material bias (Supplementary Material Table 3). Insufficient detail to make an assessment was considered an unclear risk of bias. Information that was reported but with an unclear influence on bias was also classified as unclear risk of bias. Risk of bias was assessed from the perspective of this review, which may differ from the perspective of the original study. Assessments of risk of bias were not applicable for the survey of use (objective 2) due to scope.

### Data analysis and synthesis

For the survey of use, the collected information was compiled in Microsoft® Excel and summarised in text and table form. The remaining information on data analysis and synthesis below applies to the measurement comparisons.

The identified temperature measurement comparisons were categorised according to six pre-defined concept-based outcome subgroups for presentation and synthesis (1. temperature disturbance of the surface underlying a surface sensor, 2. thermal equilibrium of the surface sensor with the underlying temperature, 3. influence of the attachment on surface sensors, 4. influence of the pressure applied by surface sensors, 5. influence of the environmental conditions on surface sensors, 6. influence of the type of surface sensor; descriptions in Supplementary Material Appendix 3). Specific details about comparisons differed from study to study (e.g., number of sensors and sites used, timing of comparison measurements) and therefore such information was retained.

Comparisons were expressed as mean temperature differences (estimate of measurement bias; calculated as comparator 2–comparator 1) and 95% limits of agreement (LoA; estimate of random error), in °C. The 95% LoA were calculated, where possible, as:

(1) $$\begin{array}{r} 95\%\text{ LoA}=\text{mean difference }\pm t_{n-1}{\cdot s}_{diff} \end{array}$$

where *t*_*n*−1_ is the corresponding critical value from the *t*-distribution, *n* is the sample size, and *s*_*diff*_ is the standard deviation of the differences. Due to being typically limited to summary data, heteroscedasticity and normality of the individual differences were assumed and not directly assessed. We used the critical value from the *t*-distribution for the calculation of LoA (cf. 1.96 or 2; Bland and Altman, 1986) because the sample sizes here were commonly <15 (Williamson et al., 2002). There were cases in which the *s*_*diff*_ was not available. In cases without any form of variance presented, the point estimates were used without LoA. In cases where other suitable parameters were reported (e.g., confidence interval for the mean difference, the standard deviation of each comparator separately), we were able to estimate *s*_*diff*_ (for further detail see Supplementary Material Appendix 4).

Studies that were unable to be presented in figures were acknowledged in narrative form (a lack of information in the original study was the typical reason). For practicality reasons, for cases in which there were more than two comparators within a specific study outcome (e.g., a comparison of seven levels of applied pressure; Jirak et al., 1975), one was designated as the “common comparator” from which the others were compared (in this example, at five body sites separately, the measured temperature with an applied pressure of 136 mmHg was compared to the measured temperature using, in series, the same sensor at the same body site with applied pressures of 34–681 mmHg). The common comparator was either dictated by the data presentation in the original article (e.g., data from Jirak et al., 1975) or it was selected by the review investigators after considering the original context of the data and the purpose of the comparison in the context of this review (e.g., data from Psikuta et al., 2014).

The interpretation of mean differences was made cognizant of the lack of a universal reference *T*_skin_ or *T*_surface_. Thus, comparisons were typically relative, without one measurement being considered more accurate (a better estimate of the so-called “true” value; an exception here is the temperature disturbance and thermal equilibrium subgroups in which the reference temperatures were assumed to be superior to the surface *T*_skin_ sensor). Similarly, the LoA were not considered to be a perfect or complete indicator of random error, but rather a reasonable and familiar estimate. The threshold for being considered practically meaningful was beyond ±0.5°C for mean difference (*in vivo* and physical models) and, for LoA, ±1.0°C for *in vivo T*skin (similar to those used elsewhere; Harper Smith et al., 2010; James et al., 2014) and ±0.5°C for physical models. These thresholds were a simplification of reality but necessary for practicality. The thresholds for human *T*_skin_ were supported by retest reliability data from contact temperature sensors (thermistors; for mean *T*_skin_ during rest and exercise in sessions separated by 1 week, mean differences were <0.5°C and LoA typically <1.0°C) (James et al., 2014). The physiological relevance of these thresholds will, in practice, depend upon a particular application (e.g., purpose of the measurement, number of sites used, the requirement for external validity of absolute values). Lower LoA thresholds were used for the physical models assuming greater experimental control of a model versus human skin. Meta-analyses were not performed due to inconsistency across studies, particularly in terms of reference condition (comparator sensor type, setup, and experimental conditions).

Data were displayed, where possible, in forest plots (Supplementary Material) and condensed into summary plots (main text). Summary plots were used for initial data display to facilitate an accessible overview while the forest plots were given in the Supplementary Material to retain the option for a more detailed examination of specific comparisons. Data for summary plots (mean, minimum, and maximum for mean differences and LoA) were calculated from the data presented in the forest plots with the mean differences first converted into absolute values (i.e., mean absolute error). Therefore, mean differences in the summary plots indicate only the magnitude of the difference, whereas mean differences in the forest plots indicate both the magnitude and relative direction.

## Results

For the measurement comparisons objective, study characteristics, assessment of risk of bias, and outcomes are presented in separate sections below. For the survey of use objective, the outcomes of interest were simply study characteristics and, therefore, all results are contained within section Study Characteristics.

### Study characteristics

**Figure 1:**
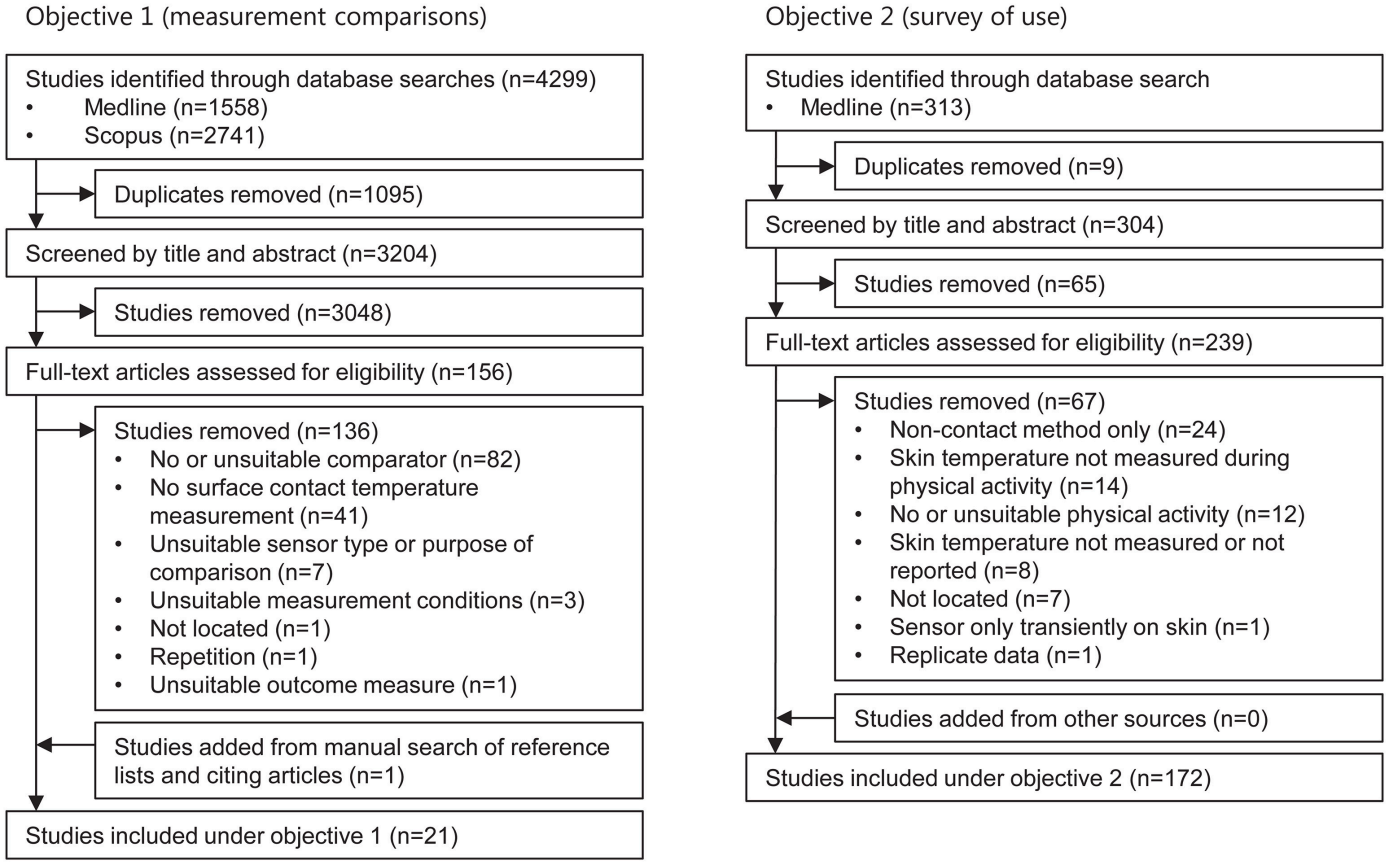
Screening flow diagram for objectives 1 and 2. Only one reason for exclusion is given per study but multiple reasons may have applied.

An overview of the literature search and study screening for the two objectives is given in Figure 1.

#### Measurement comparisons

The search yielded 4,299 records (Figure 1). Following removal of duplicates (*n* = 1,095) and exclusion during screening (*n* = 3,184), 20 studies remained. An additional study was located from searches of the reference lists. Thus, 21 studies were finally included (Yakovlev and Utekhin, 1965, 1966; Guadagni et al., 1972; Jirak et al., 1975; Flesch et al., 1976; Mahanty and Roemer, 1979a,b; Krause, 1993; Dollberg et al., 1994; Lee et al., 1994; Buono and Ulrich, 1998; van Marken Lichtenbelt et al., 2006; Deng and Liu, 2008; Harper Smith et al., 2010; Youhui et al., 2010; Tyler, 2011; James et al., 2014; Psikuta et al., 2014; Bach et al., 2015a; McFarlin et al., 2015; Priego Quesada et al., 2015). From these 21 studies, 31 distinct subsets of comparisons were identified. A subset is considered here as data from a particular study that addresses a distinct aspect of one (or more) of the outcome subgroups. Four subsets (Buono and Ulrich, 1998; van Marken Lichtenbelt et al., 2006; Harper Smith et al., 2010; Tyler, 2011) were applicable to two outcome subgroups and one study (Psikuta et al., 2014) was applicable to four, giving 38 subsets in total.

Selected general information from the included studies is given in Supplementary Material Table 4. From the 21 studies included, the relevant outcome data comprised measurements from a physical model in nine studies (Yakovlev and Utekhin, 1965, 1966; Flesch et al., 1976; Mahanty and Roemer, 1979a; Krause, 1993; Lee et al., 1994; James et al., 2014; Psikuta et al., 2014; Priego Quesada et al., 2015) and measurements from the skin of human subjects in 17 studies (Yakovlev and Utekhin, 1965, 1966; Guadagni et al., 1972; Jirak et al., 1975; Mahanty and Roemer, 1979a,b; Dollberg et al., 1994; Lee et al., 1994; Buono and Ulrich, 1998; van Marken Lichtenbelt et al., 2006; Deng and Liu, 2008; Harper Smith et al., 2010; Youhui et al., 2010; Tyler, 2011; James et al., 2014; Bach et al., 2015a; McFarlin et al., 2015). In human studies, *T*_skin_ data was reported from single body sites in 10 studies (Yakovlev and Utekhin, 1965, 1966; Jirak et al., 1975; Mahanty and Roemer, 1979a,b; Dollberg et al., 1994; Lee et al., 1994; Deng and Liu, 2008; Youhui et al., 2010; McFarlin et al., 2015) and as the mean of multiple sites in seven studies (Buono and Ulrich, 1998; van Marken Lichtenbelt et al., 2006; Harper Smith et al., 2010; Youhui et al., 2010; Tyler, 2011; James et al., 2014; Bach et al., 2015a); in two studies it was unclear if the data represented single sites or a mean of multiple sites (Yakovlev and Utekhin, 1966; Mahanty and Roemer, 1979b). The range for the number of skin sites used within a study was 1–14 (3–14 for those that reported mean *T*_skin_).

#### Survey of use

This second search yielded 313 records (Figure 1). Following removal of duplicates (*n* = 9) and exclusion during screening (*n* = 132), 172 studies were retained (see in-text Appendix). These studies included 2,267 participants (2014 male, 213 female, 40 not specified). *T*_skin_ data were reported for participants cycling in 84 studies (49% of included studies), running in 40 studies (23%), walking in 40 studies (23%), occupational PA in 7 studies (4%), and other PA in 18 studies (11%). Most studies (90%) also reported resting *T*_skin_ data.

A summary of the information about the sensors, attachments, and data use is given in Table 1. The type of contact sensor used (e.g., thermistor, thermocouple) was reported in 84% of studies. Of the studies reporting sensor type, thermistors were the most common (89 studies) followed by thermocouples (30 studies) and iButtons (oscillator-based digital thermometer; 26 studies). Identifying the sensor manufacturer (e.g., Grant Instruments Ltd, Cambridge, UK) and model (e.g., EUS-UU-VL3-0) was not always possible: in 34 studies (20%) no manufacturer or supplier information was reported and in 65 studies (38%) no sensor model information was reported. Details about sensor calibration were rarely reported with 94% of studies providing no or unclear information. Similarly, most studies (89%) reported no error-related information about the sensor (e.g., uncertainty, precision). Over half of the studies reported no information about the method of sensor attachment: of the 73 studies (42%) reporting clear information, 63 studies used tape. It was possible to determine whether the sensors were covered or uncovered in 40% of studies, with the remaining 60% unclear or not reported. Most studies reported mean *T*_skin_ (83%) and almost all studies reported absolute temperature values (97%). Irrespective of the number of measurement sites used, 57 studies (33%) reported some data from single measurement sites or single sensor.

**Table 1:** Information from survey of sensor usage in studies involving physical activity (*n* = 172 studies); data are study count with percentage in parentheses.

	**Reported**	**Unclear or not reported**
Type of contact temperature sensor	144 (84%)^a^	28 (16%)
Sensor calibration	10 (6%)	162 (94%)
Sensor attachment method	73 (42%)^b^	99 (58%)
	**Something reported**	**Not reported**
Sensor accuracy, uncertainty, precision, etc.	19 (11%)	153 (89%)
	**Covered**	**Uncovered**
Sensor coverage by attachment^c^	65 (38%)	3 (2%)
	**Yes**	**No or unclear**
*Calculations using skin temperature data*		
Mean skin temperature	142 (83%)	30 (17%)
Mean body temperature	39 (23%)	133 (77%)
Other calculations	56 (33%)	116 (67%)
	**Absolute**	**Change score**
Skin temperature data presentation	166 (97%)	30 (17%)

a *Sensor types reported were: thermistors [n = 89; the most common manufacturer reported was Grant Instruments Ltd., Cambridge, UK (n = 29) followed by YSI Inc., Yellow Springs, OH, USA (n = 18); 10 studies reported no manufacturer or supplier information], thermocouples [n = 30; the most common manufacturer or supplier reported was Concept Engineering, Old Saybrook, CT, USA (n = 5) followed by Omega Engineering Ltd, Stamford, CT, USA (n = 3); 17 studies reported no manufacturer or supplier information], iButtons (an oscillator-based digital thermometer; n = 26; Maxim Integrated Products, CA, USA), and resistance thermometers (n = 2). Three studies each used two types of contact temperature sensors so the total of sensor types here is 147 (cf. 144 in the table above)*.

b *Of the 73 reported, 63 studies used tape and 33 of those studies specified the tape type: 3 M Transpore (n = 13), 3 M Tegaderm (n = 9), BSN Medical Fixomull (n = 4), 3 M Blenderm (n = 2), 3 M Medipore (n = 2), BSN Medical Hypafix (n = 2), Hy-Tape international Hy-Tape (n = 2), Leuko Sportstape (n = 1)*.

c *The remaining (n = 104, 60%) were unclear or not reported*.

The number and location of body sites used are given in Supplementary Material Tables 5 and 6, respectively. The number of body sites per participant ranged from one to sixteen, with four sites most commonly used (46%). Chest (74%), anterior thigh (71%), lower leg (70.3%), and upper arm (56%) were the most common sites used.

### Risk of bias within studies—measurement comparisons

**Figure 2:**
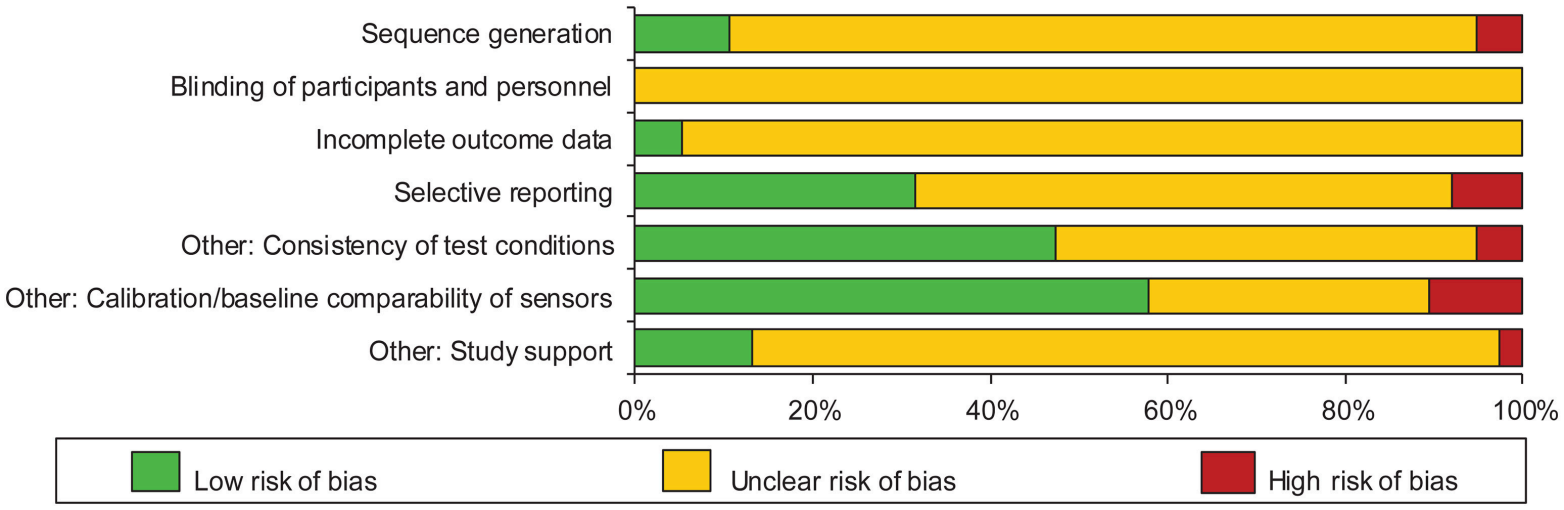
Risk of bias across all included subsets (*n* = 38 subsets).

Risk of bias for measurement comparisons was assessed at the subset level within each outcome subgroup. These assessments (given in full in Supplementary Material Figure 1) are summarised in Figure 2. In total, the risk of bias was typically unclear (72%), with only a quarter (24%) judged as low risk of bias; the remaining 5% were judged as high risk of bias. The domains most frequently judged as high risk of bias were calibration/baseline comparability of the sensors (11%) and selective reporting (8%). Making the assessment was often challenging due to limited reporting in the original articles: this limitation contributed to the high proportion judged as unclear risk of bias.

### Outcomes—measurement comparisons

The ways in which individual studies or experiments were conducted and reported were diverse and inconsistent across studies (e.g., model surfaces or skin sites used, types of sensors and attachments, environments and procedures; pooled mean or individual measurement sites or timings). Therefore, meta-analyses were not performed.

**Figure 3:**
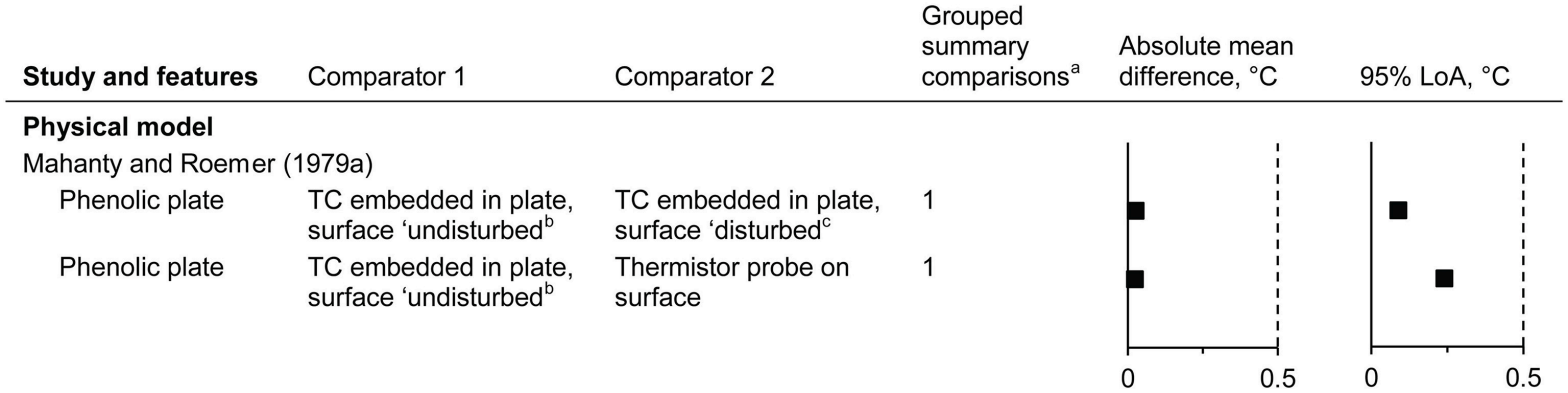
Temperature disturbance of the surface underlying a surface sensor (absolute mean difference and 95% limits of agreement). LoA, limits of agreement; TC, thermocouple. Dashed vertical lines indicate the thresholds for guiding practical significance. ^a^From the forest plots in the Supplementary Material; mean differences are presented here as absolute values, indicative of magnitude but not relative direction. ^b^Thermocouple 0.4 mm below the plate surface; temperature at the surface calculated by assuming linear variation in temperature through the plate. ^c^Temperature as in “b,” but while a surface temperature probe is in contact with the surface.

**Figure 4:**
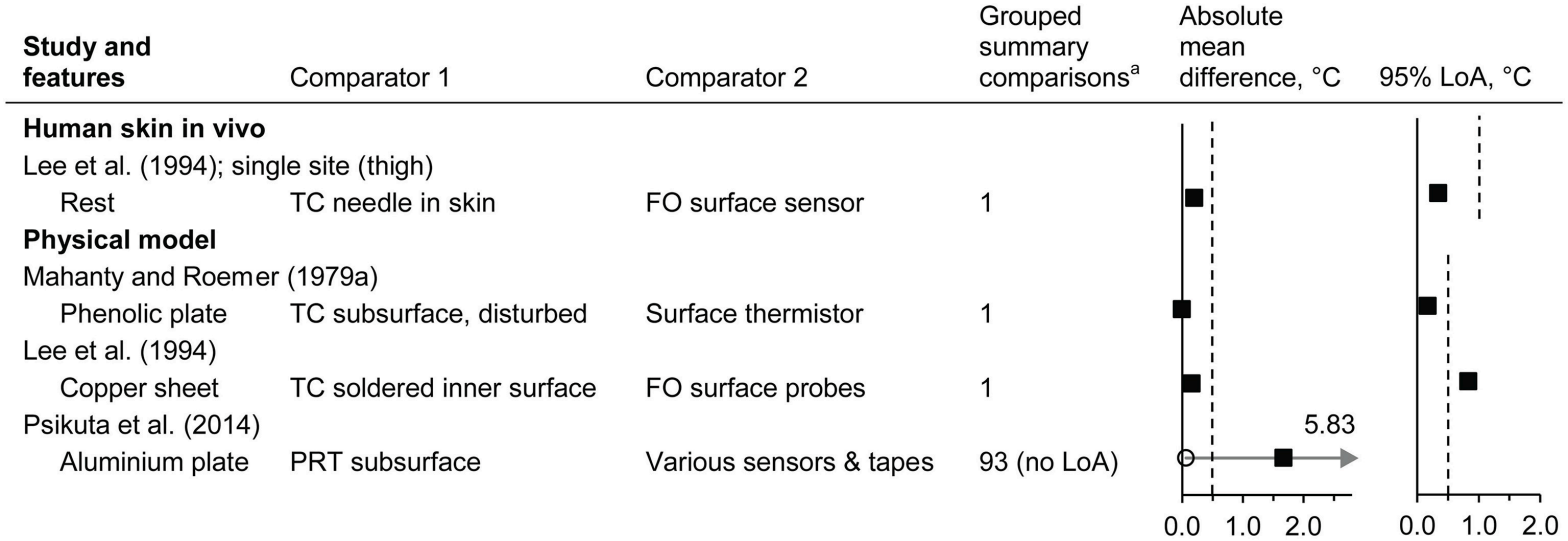
Thermal equilibrium of the surface sensor with the underlying temperature (absolute mean difference and 95% limits of agreement). FO, fibre optic; LoA, limits of agreement; PRT, platinum resistance thermometer; TC, thermocouple. Filled squares indicate mean and open circles indicate the range (minimum and maximum values). Dashed vertical lines indicate the thresholds for guiding practical significance. ^a^From the forest plots in the Supplementary Material; mean differences are presented here as absolute values, indicative of magnitude but not relative direction.

**Figure 5:**
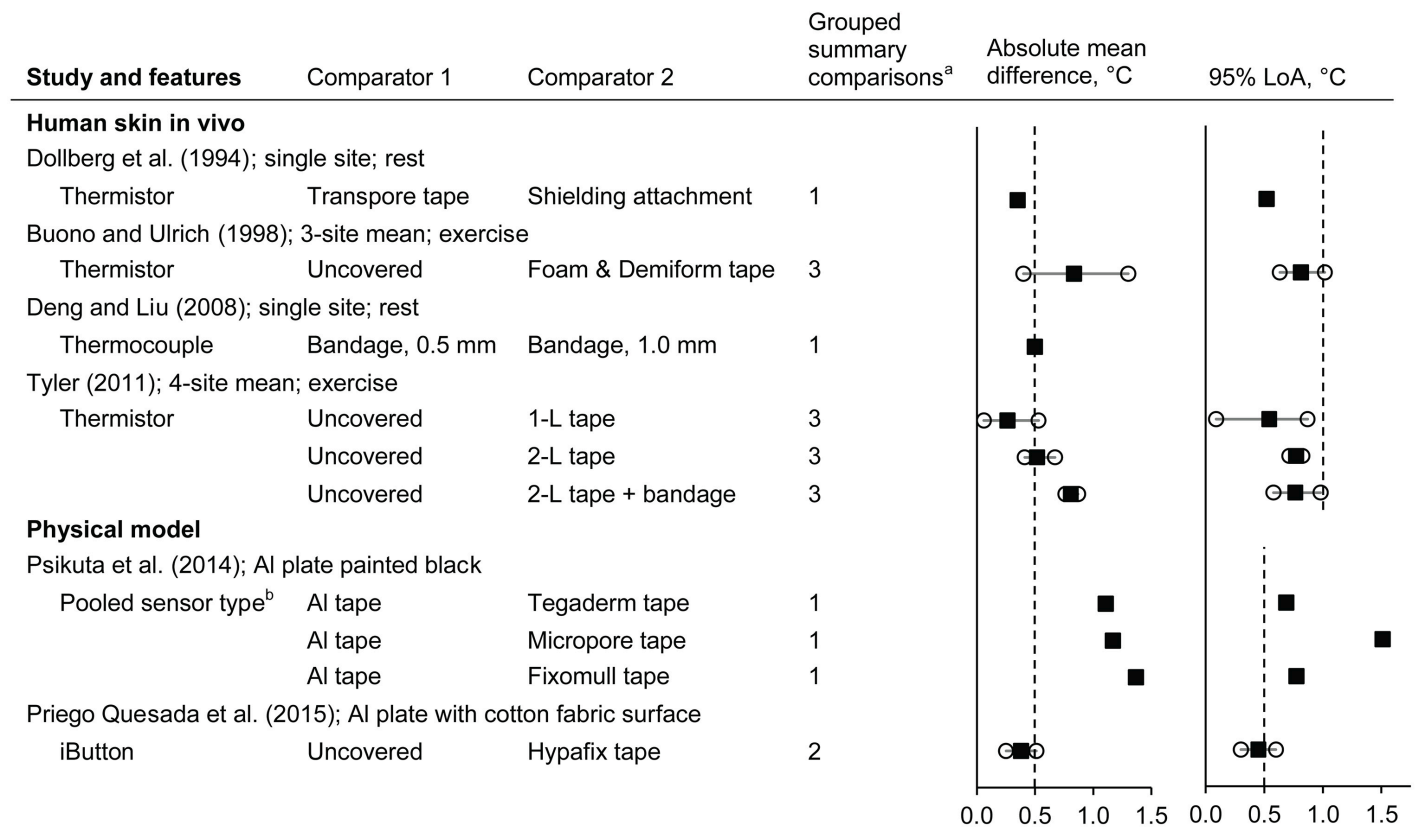
Influence of the attachment on the temperature measured by surface sensors (absolute mean difference and 95% limits of agreement). Al, aluminium; L, layer; LoA, limits of agreement; PRT, platinum resistance thermometer. Filled squares indicate mean and open circles indicate the range (minimum and maximum values). Dashed vertical lines indicate the thresholds for guiding practical significance. ^a^From the forest plots in the Supplementary Material; mean differences are presented here as absolute values, indicative of magnitude but not relative direction. ^b^PRT100 foil, thermistor, insulated PRT100, and iButton.

**Figure 6:**
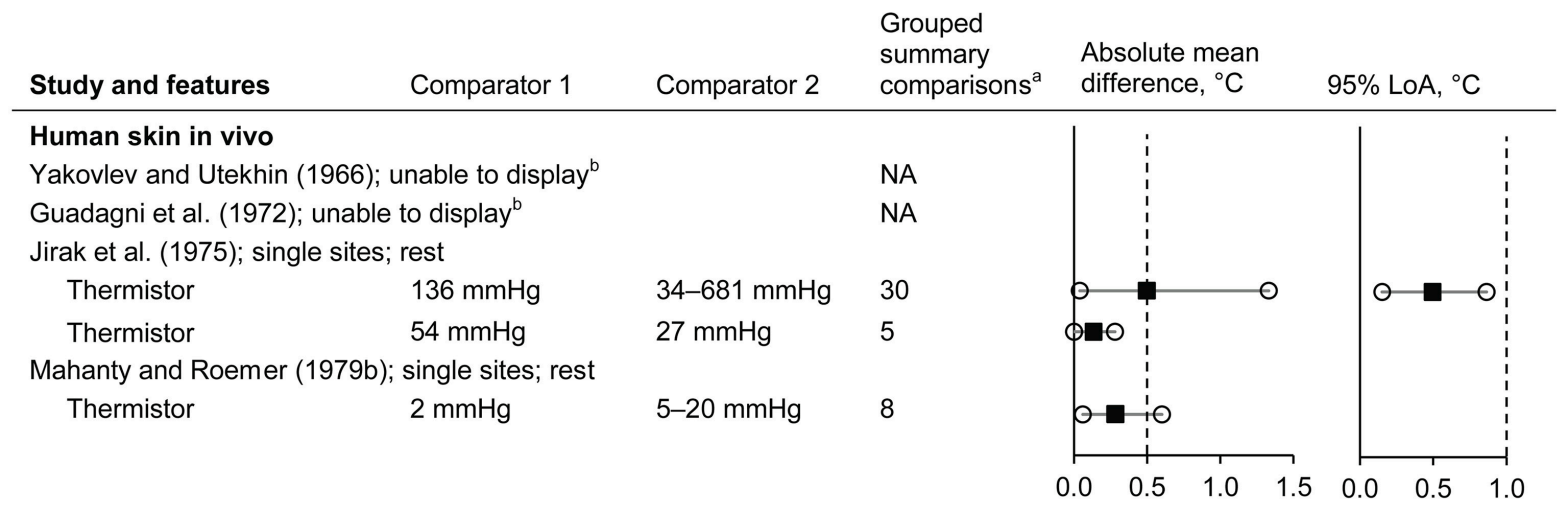
Influence of the pressure applied by surface sensors (absolute mean difference and 95% limits of agreement). LoA, limits of agreement; NA, not applicable. Filled squares indicate mean and open circles indicate the range (minimum and maximum values). Dashed vertical lines indicate the thresholds for guiding practical significance. ^a^From the forest plots in the Supplementary Material; mean differences are presented here as absolute values, indicative of magnitude but not relative direction. ^b^Not presented here due to limited detail in the original article; see text for information.

**Figure 7:**
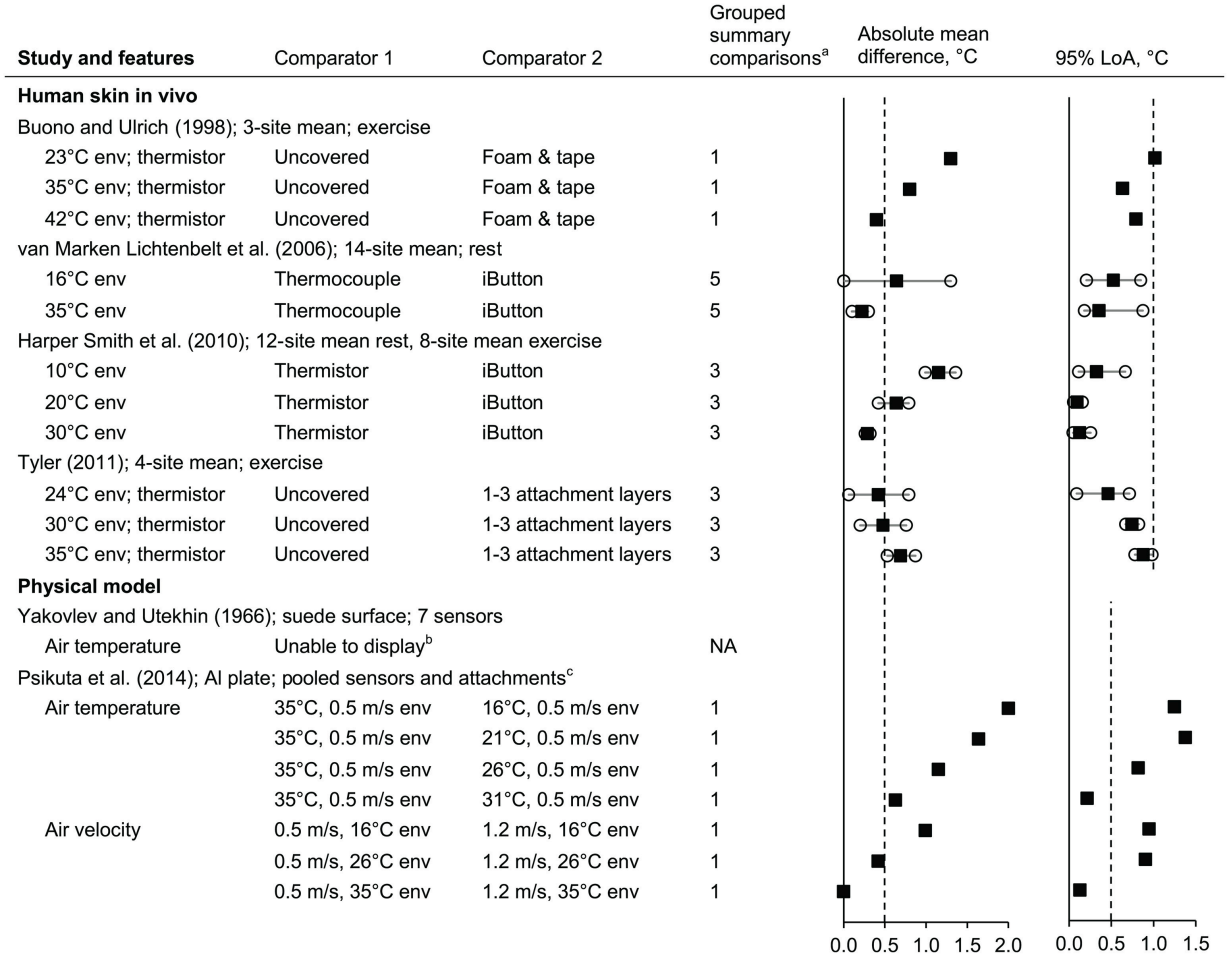
Influence of the environmental conditions on surface sensors (absolute mean difference and 95% limits of agreement). Al, aluminium; env, environment; LoA, limits of agreement; NA, not applicable; PRT, platinum resistance thermometer. Filled squares indicate mean and open circles indicate the range (minimum and maximum values). Dashed vertical lines indicate the thresholds for guiding practical significance. ^a^From the forest plots in the Supplementary Material; mean differences are presented here as absolute values, indicative of magnitude but not relative direction. ^b^Not presented here due to limited detail in the original article; see text for information. ^c^Data from all sensors (PRT100 foil, thermistor, insulated PRT100, and iButton) and attachment types (aluminium, Fixomull, Tegaderm, and Micropore tapes) are pooled here.

**Figure 8:**
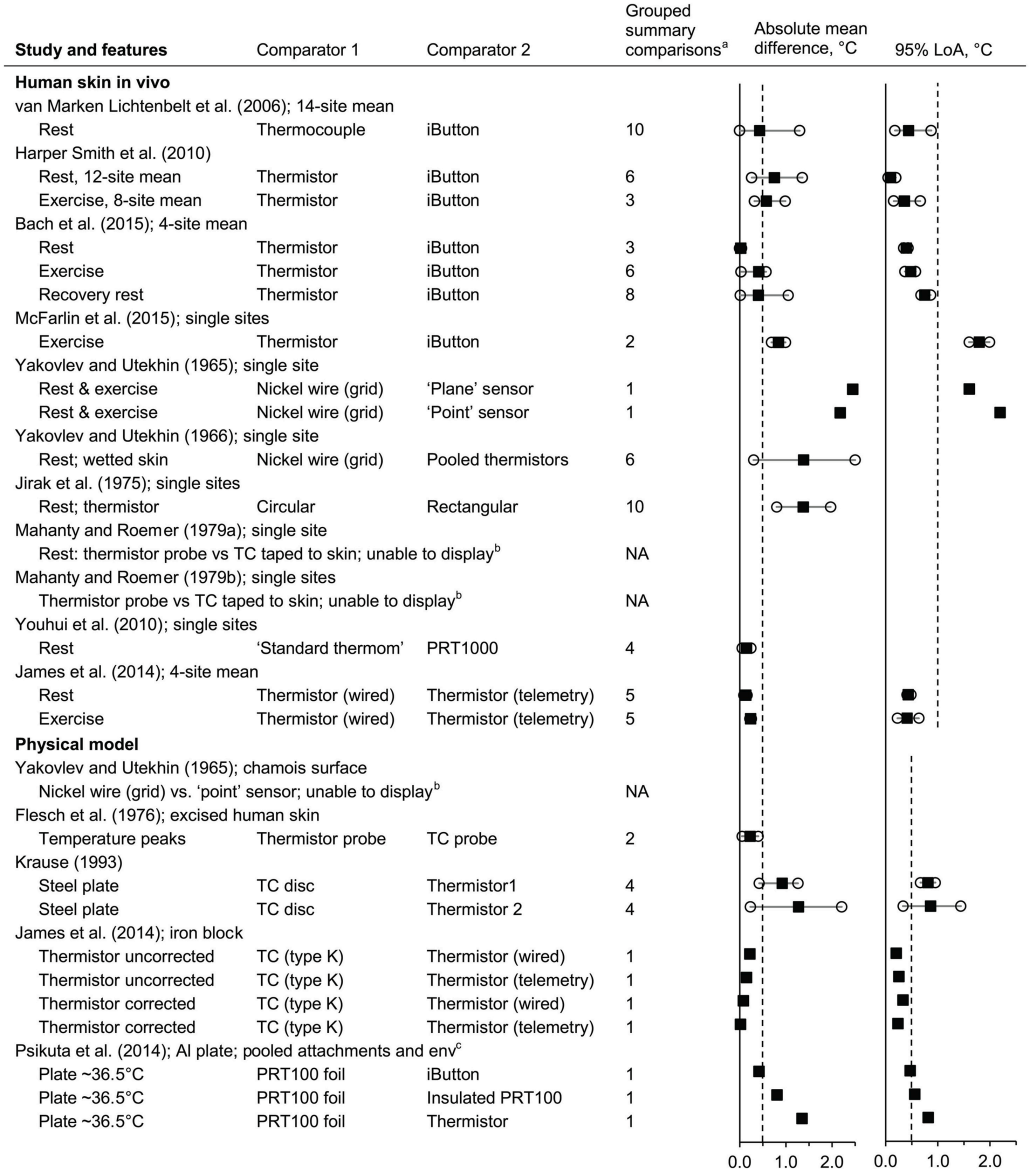
Influence of the type of surface sensor (absolute mean difference and 95% limits of agreement). Al, aluminium; env, environment; LoA, limits of agreement; NA, not applicable; PRT, platinum resistance thermometer; TC, thermocouple; thermom, thermometer. Filled squares indicate mean and open circles indicate the range (minimum and maximum values). Dashed vertical lines indicate the thresholds for guiding practical significance. ^a^From the forest plots in the Supplementary Material; mean differences are presented here as absolute values, indicative of magnitude but not relative direction. ^b^Not presented here due to the limited information reported in original article; see text for information. ^c^Data from all attachment types (aluminium, Fixomull, Tegaderm, and Micropore tapes) and environments (15–35°C, 0.5 m/s) are pooled here.

The included data are presented in summary plots (Figures 3–**8**) and corresponding forest plots (Supplementary Material Figures 2–7). Data from six subsets from five studies (Yakovlev and Utekhin, 1965, 1966; Guadagni et al., 1972; Mahanty and Roemer, 1979a,b) were unable to be presented visually due to a lack of information and, therefore, are summarised briefly in the text. The LoA were not included in seven subsets from six studies due to unreported variance estimates (Yakovlev and Utekhin, 1966; Jirak et al., 1975; Flesch et al., 1976; Mahanty and Roemer, 1979b; Deng and Liu, 2008; Youhui et al., 2010).

#### Temperature disturbance of the surface underlying a surface sensor

Data from one study were identified (Mahanty and Roemer, 1979a) (*n* = 1 subset, giving 2 comparisons; Figure 3). The surface temperature of a physical model was estimated from a thermocouple 0.4 mm below the surface, without and with a surface temperature sensor directly above (undisturbed and disturbed states, respectively). The undisturbed surface temperature was similar to both the disturbed temperature and the surface thermistor probe acting as the disturbance (mean differences of <0.1°C for the point estimates).

#### Thermal equilibrium of the surface sensor with the underlying temperature

Data from three studies were identified (Mahanty and Roemer, 1979a; Lee et al., 1994; Psikuta et al., 2014) (*n* = 4 subsets, giving 96 comparisons; Figure 4 and Supplementary Material Figure 3). Temperature of the externally applied surface sensors was typically lower than invasive *T*_skin_ *in vivo* (Lee et al., 1994) and lower than the model reference temperature (Lee et al., 1994; Psikuta et al., 2014), although the magnitude of the mean differences was variable, ranging from <0.5°C (Mahanty and Roemer, 1979a; Lee et al., 1994; Psikuta et al., 2014) to 5.8°C (Psikuta et al., 2014). For surface *T*_skin_ sensors and attachments commonly used in non-clinical human research studies (Psikuta et al., 2014), greater differences were observed with a greater surface-environment gradient (see also influence of the environmental conditions). The data from Krause (1993) was not included here because the reference temperature sensors within the physical model are not described and no plate temperature data is reported.

#### Influence of the attachment on surface sensors

Data from six studies were identified (Dollberg et al., 1994; Buono and Ulrich, 1998; Deng and Liu, 2008; Tyler, 2011; Psikuta et al., 2014; Priego Quesada et al., 2015) (*n* = 6 subsets, giving 19 comparisons; Figure 5 and Supplementary Material Figure 4). The type of attachment used had mixed effects on the mean differences of *T*_skin_ or *T*_surface_ with absolute mean differences ranging from 0.1 to 1.4°C. The LoA were typically within ±1.0°C for measurements on human skin, while those on physical models ranged from 0.3 to 1.5°C.

Using an attachment resulted in numerically greater measured temperatures by 0.1–1.3°C compared to the same sensors “uncovered” for human skin (Buono and Ulrich, 1998; Tyler, 2011) or a model surface (Priego Quesada et al., 2015). With increasing environmental temperature, the differences of covered versus “uncovered” sensors became smaller in one study (mean difference of 1.3°C in a 23°C environment, decreasing to 0.4°C in 42°C) (Buono and Ulrich, 1998) but tended to become larger in another study (mean difference of 0.1°C increasing to 0.5°C for one layer of tape, 0.8°C increasing to 0.9°C for two layers of tape plus bandage, environments of 24° and 35°C, respectively; Supplementary Material Figure 4) (Tyler, 2011). Shielding the sensor (Dollberg et al., 1994), increasing the attachment thickness (Deng and Liu, 2008), or increasing the number of attachment layers (Tyler, 2011) tended to increase the measured temperature. One study with pre-term infants inside incubators had a pertinent risk of bias in that the measured *T*_skin_ itself influenced the environmental conditions within the incubator (Dollberg et al., 1994). In that study, higher measured temperature caused lower environmental temperatures and so this risk of bias likely mitigated the magnitude of the observed difference: indeed, the net effect was a 0.8°C greater skin-to-environment temperature gradient when the shielding attachment was used. Elsewhere, aluminium tape typically resulted in higher measured temperatures than other common surgical tapes (>1°C for pooled mean differences), although interestingly, these higher *T*_surface_ were typically closer to the expected *T*_surface_ of the aluminium plate (Psikuta et al., 2014) (see also section Thermal Equilibrium of the Surface Sensor with the Underlying Temperature).

#### Influence of the pressure applied by surface sensors

Data from four studies were identified (Yakovlev and Utekhin, 1966; Guadagni et al., 1972; Jirak et al., 1975; Mahanty and Roemer, 1979b) (*n* = 4 subsets; 2 subsets displayed, giving 43 comparisons; Figure 6 and Supplementary Material Figure 5). Absolute mean differences ranged from 0 to 1.33°C for the pressure comparisons available (Figure 6). The measured temperature tended to increase with increasing pressure over the range of 2–681 mmHg (Jirak et al., 1975; Mahanty and Roemer, 1979b). Halving or doubling the applied pressure resulted in mean differences consistently within ±0.5°C (note that the common comparison pressures were used as presented in the original articles) (Jirak et al., 1975; Mahanty and Roemer, 1979b). For example, for a circular sensor at the forehead the mean differences, versus 136 mmHg, were −0.31°C at 68 mmHg and +0.36°C at 272 mmHg (Jirak et al., 1975; Supplementary Material Figure 5). From the data of one subset (Jirak et al., 1975), the LoA were typically greater when the higher pressures were included (409, 545, and 681 mmHg), although all within ±1.0°C.

One of the subsets not able to be displayed in the plots (Guadagni et al., 1972) was simply summarised in the original article as a statement that the effect of pressure on steady state skin temperature (human participants; sites not reported) was <0.01°C at pressures between 31 and 52 mmHg, and that at pressures beyond this range, the steady state temperature becomes pressure-dependent and increases with increasing pressure. For the other subset not able to be displayed (Yakovlev and Utekhin, 1966), differences of 0.1–0.7°C were reported for the seven sensor types tested on human skin (sites not reported) whereby the pressure was generated by 5 and 70 g weights (sensor surface areas were not reported, therefore the pressures are unknown). Notwithstanding, the authors did state, without the supporting data, that pressure effects can be disregarded when the maximum pressure of the sensor on the skin does not exceed 15–37 mmHg.

#### Influence of the environmental conditions on surface sensors

Data from six studies were identified (Yakovlev and Utekhin, 1966; Buono and Ulrich, 1998; van Marken Lichtenbelt et al., 2006; Harper Smith et al., 2010; Tyler, 2011; Psikuta et al., 2014) (*n* = 6 subsets; five subsets displayed, giving 38 comparisons; Figure 7 and Supplementary Material Figure 6).

For the subsets in which human participants were used (Buono and Ulrich, 1998; van Marken Lichtenbelt et al., 2006; Harper Smith et al., 2010; Tyler, 2011), potential influences of the environment can be examined indirectly via the relative differences for the same two comparators under different environments—here, the different environments cannot be directly compared because the *T*_skin_ will also change. (Because these indirect comparisons may be confounded by other variables such as the differences in absolute *T*_skin_, they are interpreted cognizant of this possible but unclear risk of bias.) Irrespective of the underlying cause, the mean difference between the same two comparators were often different when the comparison was made under different environmental temperatures in three subsets (Buono and Ulrich, 1998; van Marken Lichtenbelt et al., 2006; Harper Smith et al., 2010): for example, relative mean differences of 1.3 and 0.4°C for the same attachment comparison under 23 and 42°C environmental temperatures (Buono and Ulrich, 1998). In other words, the magnitude of mean difference for a given comparison of sensor setups showed some dependence on the environmental temperature. For the influence of air movement during rest, the pattern of relative differences at 10, 20, and 30°C environmental temperatures was similar under both 0.2 and 2.3 m/s air velocities (e.g., relative mean differences of 0.70 and 0.79°C for the two velocities, respectively, in a 20°C environment; Supplementary Material Figure 6) (Harper Smith et al., 2010).

For one subset in which a model was used (Psikuta et al., 2014), data are presented in Figure 7 as pooled estimates for main effects of the environmental variables. In this subset (Psikuta et al., 2014), compared to *T*_surface_ measurements in the 35°C environment, the cooler environments resulted in lower, and more variable, measured values despite the plate being maintained at ~36.5°C; the magnitudes of mean differences were often of practical relevance (>0.5°C). Comparing wind velocity within the same environmental temperature indicates that the influence of an increase in wind velocity (from 0.5 to 1.2 m/s) was negligible when the environmental temperature (35°C) was close to the *T*_surface_ (36.5°C) but became increasingly relevant as the environmental temperature diverged further from the plate temperature (relative differences of −0.4 and −1.0°C for the point estimates in environments of 26 and 16°C, respectively). Data from the other subset using a physical model are not presented due to uncertainties in interpretation of the environment and the reference *T*_surface_ but the authors did state effects of the ambient air temperature being 0.2–6.0°C for seven different sensor types (Yakovlev and Utekhin, 1966).

#### Influence of the type of surface sensor

Data from 14 studies were identified (Yakovlev and Utekhin, 1965, 1966; Jirak et al., 1975; Flesch et al., 1976; Mahanty and Roemer, 1979a,b; Krause, 1993; van Marken Lichtenbelt et al., 2006; Harper Smith et al., 2010; Youhui et al., 2010; James et al., 2014; Psikuta et al., 2014; Bach et al., 2015a; McFarlin et al., 2015) (*n* = 17 subsets; 14 subsets displayed, giving 87 comparisons; Figure 8 and Supplementary Material Figure 7). The risk of bias for calibration of these sensors used was considered high for four subsets [*in vivo* (McFarlin et al., 2015) and models (Flesch et al., 1976; Krause, 1993), (James et al., 2014) model uncorrected], unclear for five subsets [*in vivo* (Yakovlev and Utekhin, 1965, 1966; Mahanty and Roemer, 1979a; Youhui et al., 2010), and model (Yakovlev and Utekhin, 1965)], and low for eight subsets [*in vivo* (Jirak et al., 1975; Mahanty and Roemer, 1979b; van Marken Lichtenbelt et al., 2006; Harper Smith et al., 2010; James et al., 2014; Bach et al., 2015a) and models (Psikuta et al., 2014; James et al., 2014) model corrected].

Absolute mean differences for some subsets were almost consistently within (Youhui et al., 2010; James et al., 2014) or beyond 0.5°C (Yakovlev and Utekhin, 1965, 1966; Jirak et al., 1975; Krause, 1993; McFarlin et al., 2015). For four *in vivo* studies, the LoA were almost consistently within ±1.0°C (van Marken Lichtenbelt et al., 2006; Harper Smith et al., 2010; James et al., 2014; Bach et al., 2015a) and in two studies consistently greater than ±1.0°C (Yakovlev and Utekhin, 1965; McFarlin et al., 2015).

Data from three subsets were not able to be presented in the plots due to insufficient information [human participants at rest (Mahanty and Roemer, 1979a,b) and a model (Yakovlev and Utekhin, 1965)]. According to the text of the original articles, a thermocouple taped to the skin and a thermistor probe “agreed within ±0.15°C (maximum difference)” on one occasion (Mahanty and Roemer, 1979a) and had a mean difference of 0.03°C on the other occasion (Mahanty and Roemer, 1979b). For the physical model (Yakovlev and Utekhin, 1965), a nickel wire sensor and a “point” sensor (diameter 0.3–1 mm) were compared following the wetting of the suede surface of a model: the temperature from the nickel wire sensor decreased by approximately 4°C over the subsequent 2 min whereas the temperature from the “point” sensor decreased by <1°C over the same period.

## Discussion

In comparison measurements from various contact *T*_skin_ sensor setups—using human participants or relevant physical models—mean differences exceeding 0.5°C were observed for some comparisons of attachment, applied pressure, and sensor type. Additionally, there was indication that the environmental conditions can influence the measured temperature and that the surface sensor is not always sufficiently at thermal equilibrium with the underlying surface. Thus, the sensor type used and how it is used can meaningfully influence the measured value. For users of contact *T*_skin_ sensors, emphasis should be placed on consistency of sensor setup parameters (at least within a study) and the limitations of knowing an absolute *T*_skin_ must be recognised, particularly for comparability across studies. The survey of contact *T*_skin_ measurements here illustrates that, for the range of sensor setups used in different studies involving sport, exercise, and other PA, important information about features like calibration and the attachment method are often unreported. For the continuing improvement of research and clinical measurements, and with growing commercial and consumer interest in wearable technology and personal body monitoring, this review demonstrates that even routine measurements are not necessarily as simple as they may otherwise seem.

### Measurement comparisons

#### Strengths and limitations

This is the first systematic review to comprehensively collate data from comparisons of measurements made using contact *T*_skin_ sensors. A strength of this work is the breadth of information included and the ability to examine specific outcomes of interest in detail.

While comparisons of outcome data were typically possible, meta-analyses were not performed due to the appreciable variety of test conditions, sensor setups, and types of temperature data used (e.g., single site versus mean *T*_skin_; multiple measurements within a period versus period grand mean), and the resultant lack of a common reference condition. Further, the lack of a universal gold-standard method for measuring *T*_skin_ limited the ability to judge one comparator *T*_skin_ sensor setup as being more accurate than the other. For this reason, emphasis was typically placed on the magnitude of effects rather than the direction of the effect.

The often limited reporting of methodological information contributed to the number of “unclear” judgements within the risk of bias assessments and made extraction of study information or data challenging at times. To guide both study design and reporting of study information in future studies comparing temperature measurements, we recommend authors to prospectively think about how their work will be judged during a subsequent risk of bias assessment. Details to consider include—but are not limited to—sequence generation, any blinding, completeness of outcome data (e.g., detachment of sensors, sensor malfunctions), selective reporting (data that was intended to be reported but was not), consistency of test conditions, sensor calibration details, and study support.

We used threshold values to indicate practical significance throughout this review (human skin *in vivo*, ±0.5°C for mean difference and ±1.0°C for 95% LoA; physical models, ±0.5°C for mean difference and 95% LoA). This was a necessary simplification for feasibility and is intended as a general guide only. It is likely that, in practice, these thresholds will need to be adjusted according to specific measurement contexts and objectives. For example, multiple spot measurements from contact *T*_skin_ sensors are often used to calculate a mean *T*_skin_, ostensibly representative of the body as a whole. Temperature differences due to the sensor setup itself may in some cases be within differences due to other experimental decisions such as site selection, replication of sensor placement, or the weightings used (Livingstone et al., 1987; Choi et al., 1997).

It is acknowledged that additional descriptive statistics are also useful for making inferences in method comparisons (e.g., typical error of the estimate, regression and correlation coefficients). Finally, not considered here was any influence of the signal processor/data logger setup (Jutte et al., 2005).

#### Validity of the surface measurement

**Figure 9:**
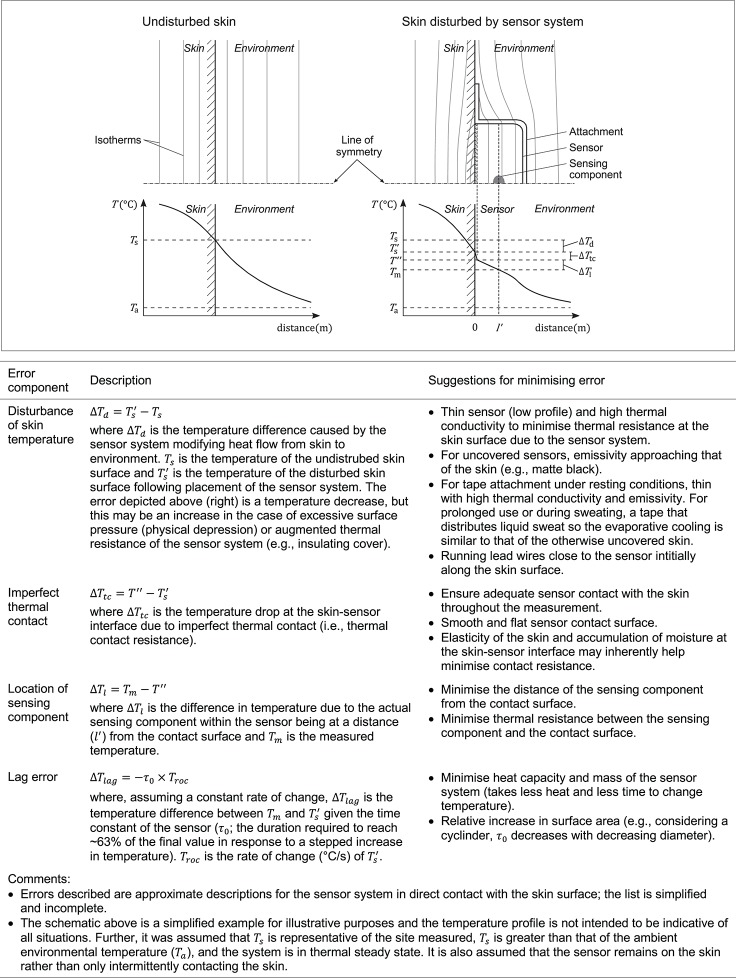
Measurement of skin temperature using a contact sensor attached to the skin surface is associated with a number of potential sources of error, some of which are illustrated and described here (Michalski et al., 2001; Nicholas and White, 2001). The schematic represents, in the case of undisturbed skin (left) and disturbed skin (right), cross sections with hypothetical isotherms (top) and the corresponding temperature profiles at the line of symmetry (bottom).

While the skin surface is an accessible site for measurement, it presents difficulties for accurate quantification of temperature in that the surface is an interface between distinct mediums, with each medium having its own thermal properties and temperature gradients. Accordingly, the sensor system is in partial contact with medium of interest (i.e., the skin) and partial contact with the adjacent environment (e.g., microclimate air, liquids, clothing). Accurate knowledge of *T*_skin_ requires the sensor temperature to be in equilibrium with the corresponding skin temperature and for the sensor setup itself to have negligible additional effect on heat exchanges between the underlying skin and its adjacent environment(s) (Bedford and Warner, 1934; Hardy, 1934b; Childs, 2001; Taylor et al., 2014). Demonstrable differences among various *T*_skin_ sensor setups may reduce, at least in part, to how well each respective sensor setup achieves this balance between thermal equilibrium and temperature modification. Selected sources of error that may be expected during *T*_skin_ measurements are given in Figure 9, along with suggestions for how such an error may be minimised (Michalski et al., 2001; Nicholas and White, 2001). Considering sources of error and error minimisation can assist *T*_skin_ sensor design or assist sensor selection for those measuring *T*_skin_. Notwithstanding, ideal dimensions or thermal properties of sensors and their attachments cannot govern sensor design or setup selection alone as these devices must also be robust and suitable enough for practical use (Youhui et al., 2010; Webb et al., 2013).

The data identified within this review were limited and incompletely address the question of validity of common *T*_skin_ sensors and so future work is warranted. In particular, estimates of uncertainty during *T*_skin_ measurements and the magnitude of any effects of the sensor and attachment disturbing the underlying *T*_skin_ are unclear. From data of the only study identified, there was no demonstrable effect of thermal disturbance of the site underlying a surface sensor (Mahanty and Roemer, 1979a). However, it is unlikely this finding is generalizable to conventional *T*_skin_ sensor types and across environmental conditions as the surface probe used was designed specifically to minimise any such modification of *T*_surface_ and the environmental temperature used (~22°C) represented a moderate difference from the surface temperature (29–33°C). Notwithstanding, this finding does illustrate the promise of sensor design based on error minimisation (Figure 9). Key characteristics of this sensor included using a small sensing component (thermistor) housed in a stainless steel disc (19 mm diameter, 0.13 mm thick), which was partially painted matte black (high emissivity, similar to skin) and partially covered with reflective silver coating to compensate for addition heat losses due in part to the surface area of the sensor system being greater than that of the skin itself (Mahanty and Roemer, 1979a).

Numerical modelling has indicated that insulated *T*_skin_ sensors can cause the temperature of the underlying skin to be higher than it would otherwise be because insulating the sensor from the adjacent environment also insulates the underlying skin (Boetcher et al., 2009). On the contrary, sensors with high thermal conductivity and no insulating layer can lower the underlying *T*_skin_ by inducing a fin effect whereby the total surface area of the sensor exposed to the environment is greater than that of the skin it covers and, in this way, heat conducted from the skin to the sensor can be lost by convection to the adjacent environment at a rate that exceeds that of the undisturbed skin itself (Guadagni et al., 1972; Boetcher et al., 2009). Heat transfer from the sensor to the environment by radiation may be greater or lesser than that of the undisturbed skin because, despite the greater surface area available for radiating heat in the case of the sensor, the emissivity of the skin is itself very high (~0.98; Steketee, 1973).

The skin has a boundary layer of air that mitigates the temperature difference directly adjacent to its surface. This difference under warm-hot conditions (relatively still air, ambient temperature of 28–29°C) may be <0.5°C for air within 1 mm, and <1°C for air up to 6 mm from the skin surface (McGlone and Bazett, 1927). In cooler conditions or with forced air convection (e.g., wind or body movements), this gradient increases and the temperature difference can become appreciable within the height of commonly used *T*_skin_ sensors and their attachments. For surface *T*_skin_ sensor equilibrium with the underlying temperature, a range of magnitudes was observed from <0.5°C (Mahanty and Roemer, 1979a; Lee et al., 1994; Psikuta et al., 2014) up to 6°C (Psikuta et al., 2014). However, details about the ambient environment were not clear from the two of the studies included here (Mahanty and Roemer, 1979a; Lee et al., 1994).

Psikuta et al. (2014) observed that ambient temperature, and thus the surface-environment gradient, can influence the estimated error: while few sensor-attachment combinations were within 1°C of the plate temperature in the 16°C environment, all were within this range in the 35°C environment. The temperature of the aluminium plate was reported as 36.5±0.03°C in this experiment and, therefore, it is intuitive that errors tend to reduce as the ambient temperature approaches the surface temperature.

Although numerical modelling and non-contact methods can also add insight, it was not feasible to also cover these in detail here and contact versus non-contact methods have been discussed elsewhere (Bach et al., 2015b). As mentioned previously, numerical modelling has indicated that, at a simulated environmental temperature of 22°C and *T*_skin_ of ~31.7°C, errors approaching 0.5°C can be expected with insulated contact sensors (Boetcher et al., 2009). Numerically modelling performed by Deng and Liu (2008) indicated that, for the attachment material, decreasing the thickness and increasing the thermal conductivity decreased the measurement error. These results are consistent with experimental observation of aluminium tape tending to better reflect the underlying plate temperature, particularly at low wind velocities (0.5 m/s) (Psikuta et al., 2014). For improving absolute temperature measurement, more work is required to delineate measurement errors in the context of human skin, especially during sweating.

#### Comparisons of setup variables

Sensor setup variables can influence the measured values from *T*_skin_ sensors. Examples of both small and practically significant effects (less than or greater than 0.5°C absolute mean difference, respectively) can be found for the outcome subgroups of attachment type, sensor-applied pressure, environmental conditions, and sensor type. The 95% LoA were often, though not always, within ±1.0°C for human skin *in vivo* and ±0.5°C for physical models. An implication of these observations is that equivalent measurements of *T*_skin_ cannot be assumed when different components of the sensor setup or different environments are used. Thus, these setup variables (e.g., sensor type, attachment, environmental conditions) should be considered prior to measurement and clearly reported to assist subsequent interpretation of *T*_skin_ measurements. Further work could identify or experimentally justify particular setups or conditions in which measurement biases are mitigated (Figure 9).

Relative temperature differences between sensor types can vary with the environmental temperature, activity, and time (van Marken Lichtenbelt et al., 2006; Harper Smith et al., 2010; Bach et al., 2015a), although this is not always the case (James et al., 2014). In other words, two different setups may not simply be a consistent offset from each other, but rather vary in magnitude with other parameters. Physical properties of the sensors and their attachments likely contribute to the consistency, or inconsistency, of relative differences, particularly during transients.

Some patterns in temperature differences during changes in environmental temperatures could reflect an influence of thermal inertia (Supplementary Material Figure 7A): iButtons appeared to either have a reduced response time to a true change in *T*_skin_ or, alternatively, they were less influenced by the change in environment and better reflected the true change in *T*_skin_ (van Marken Lichtenbelt et al., 2006; Bach et al., 2015a). The practical consequences due to the thermal inertia of the sensor setup will likely depend on the time course of the expected change in *T*_skin_. The response time of the sensor on the skin surface should be a key consideration when relatively fast changes in *T*_skin_ are expected (Arunachalam et al., 2008), but may be of lower importance compared to other considerations when a relatively stable *T*_skin_ is expected. In research settings it is common—and good practice—for participant instrumentation to occur well before experimental recordings are made, which should mitigate any transient effects of initial sensor temperature before application and the duration of a sensor to reach a thermal steady state once applied (Guadagni et al., 1972).

Surface pressure is known to influence skin perfusion, however, studies here indicated that the measured *T*_skin_ temperature almost invariably increased with increasing pressure whereas skin perfusion tends to decrease, even with a little as 5 mmHg (Holloway et al., 1976). Physical effects of the surface sensor pressing into the skin were investigated further by Mahanty and Roemer (1979b) using numerical modelling and the calculated effects agreed closely with their experimental results, suggesting these physical factors associated with depression are a suitable explanation. While increasing the sensor-applied pressure may lead to temperature errors, the thermal contact must also be sufficient and, therefore, there is likely a trade-off between the two. Unstable temperature readings were attributed to insufficient thermal contact at pressures lower than 31 mmHg in one study (Guadagni et al., 1972), but such instability issues were not apparent in another study investigating pressures of 2–20 mmHg (Mahanty and Roemer, 1979b). In the latter study, the authors suggested that 2 mmHg was sufficient for measuring *T*_skin_ and used numerical modelling to support this observation (Mahanty and Roemer, 1979b).

Controlling the applied pressure is a challenge for measuring *T*_skin_ *in vivo*. While sensor-applied pressure variations during normal use are not clear, it is notable that mean differences in *T*_skin_ due to halving or doubling a given applied pressure were <0.5°C. This finding was consistent across the wide range of pressures investigated (2–681 mmHg). Increasing the sensor surface area can be considered with respect to limiting the applied pressure and associated depression into the skin, but any such increases in area may have consequences for modifying evaporative and non-evaporative heat exchanges between the skin and the environment.

A challenge for measurement comparisons on human skin is that *T*_skin_ varies by time and location (Pennes, 1948; Kaufman and Pittman, 1966; Webb, 1992). Possible comparisons on human skin include using identical measurement sites at different times, or simultaneous measurements at different sites. While some authors compare results from single sites, others use multiple measurement sites and compare the resultant mean *T*_skin_. With respect to temporal changes in *T*_skin_ of human participants, Guadagni et al. (1972) observed that after 2.5–3 h rest in a room temperature of 24.5°C, changes in *T*_skin_ at a given site were <0.01°C within 2 or 3 min periods. A different approach was used by Mahanty and Roemer (1979b) whereby throughout a measurement period involving different experimental sensor-applied pressures, adjacent temperature sensors remained in place to allow minor temperature changes not due to experimental sensor pressure to be compensated for. Irrespective of the approach, care needs to be taken to minimise risks of systematic temperature bias.

With the expanding interest in wearable technology for day-to-day applications, there should be an expectation of acceptable validity for any measurements being taken (Sperlich and Holmberg, 2017). A direct outcome here is that sensor location and the effects of the carrier devices or attachments will need to be taken into account for such applications.

### Survey of use

#### Strengths and limitations

Here we have collected information about how contact *T*_skin_ sensors are currently (2011–2016) used and reported in published studies involving sport, exercise and other PA. Thus, this work provides context and a basis for integrating the findings delineated above in more challenging conditions than rest. These findings may, however, only provide limited information on these aspects in other research or clinical conditions.

#### General implications

In human studies, details about *T*_skin_ sensor setup details were often poorly reported and, from those reporting setup information, it was evident that the setups often varied in terms of the sensors, attachments, and locations used. Key setup variables need to be considered further and consistently reported. For example, the sensor attachment method was clear in only 42% of the articles sampled yet attachment can bias the measured values (Buono and Ulrich, 1998; Psikuta et al., 2014). Similarly, clear information about sensor calibration was present in only 6% these studies.

Absolute *T*_skin_ values are used often in research with 97% of the studies sampled here reporting some form of absolute *T*_skin_ and 17% reporting change scores (some reported both, but not necessarily for all the data). While common inferential statistical approaches end up treating absolute values as relative differences (e.g., within-subject differences for repeated measures, difference between group means for independent samples), the point remains that absolute values are of interest to researchers and other users of *T*_skin_ information. Thus, clearer reporting of setup variables may also improve awareness about the external validity of published absolute *T*_skin_ data, such as for inter-study comparisons or applied use of *T*_skin_ (e.g., temperature thresholds during heat stress).

Sensors used in the comparisons of measured temperatures (objective 1) only partially represented the sensors currently used in human studies involving PA. The outcome of some comparisons may be more generally applicable, such as effects of the sensor pressure, whereas the outcomes of other comparisons may be restricted to specific setups or environments, such as sensor type or a particular skin-environment temperature gradient. A reason contributing to specificity of comparison outcomes is that different sensor setups likely vary in variables not taken into account here, such as sensor and attachment composition (and resultant thermal properties), dimensions, and surface contact. Future work will benefit from the identification of setup variables suitable for widespread use.

### Summary

This work serves to establish a base from which users of contact *T*_skin_ measurements can make better-informed decisions about setup of the sensor-attachment system with relevance to their particular measurement objectives and the expected measurement conditions. Some potentially relevant components within the measurement system are summarised in Table 2.

**Table 2:** Summary of some relevant considerations for the measurement of skin temperature.

**Component of system**	**Consideration for component**
Skin	Selection of sites and consistency of placement Homogeneity of surface skin temperature Temperature gradients within the skin itself Lateral heat transfer from skin adjacent to the site being measured Sweating Sensor pressure (depression into the skin)
Skin-sensor interface	Thermal contact resistance Contact surface area
Sensor	Physical dimensions (including surface area of the sensor versus that of the skin site covered) and thermal properties (including the resultant thermal resistance) Obstruction of evaporative heat loss from skin Calibration method and range
Attachment	Physical dimensions and thermal properties (including thermal resistance and water vapour resistance) For tapes covering the sensor, emissivity and wetting characteristics for distributing liquid sweat Effectiveness of remaining in place during prolonged use or physical activity
Clothing or coverings and associated microclimate	Present or not Temperature gradients (microclimate temperature will typically be closer to skin temperature where clothing/coverings used than without) Air velocity Humidity
Wider environment for uncovered sites	Temperature gradients (difference from skin temperature) including air temperature and radiant temperature Air velocity Humidity

*This list is not complete and not all of the factors will be relevant in each case*.

For those performing studies comparing different *T*_skin_ sensors or sensor setup variables (e.g., method agreement studies), key points of consideration include:

- There is currently no universally applicable gold-standard *T*_skin_ measurement, which likely contributes to the general lack of a common reference condition across studies. In lieu of a specific sensor setup available to be used consistently among studies, key features of the setups used need to be described. This information includes the sensor manufacturer and model, type (e.g., thermistor), some physical characteristics (e.g., insulated or uninsulated, physical dimensions), attachment, and calibration details.
- We recommend that authors consider criteria used in risk of bias assessments to help guide study design and reporting. This information includes sequence generation, any blinding, completeness of outcome data (e.g., detachment of sensors, sensor malfunctions), selective reporting (data that was intended to be reported but was not), consistency of test conditions, sensor calibration details, and study support.
- There is scope for future work to better clarify the measurement validity of commonly used *T*_skin_ sensor setups, particularly under conditions in which the skin-to-environment gradient becomes large and when relatively fast changes in *T*_skin_ are expected. Practical effects of surface *T*_skin_ sensors on modification of heat and mass transfer from the skin surface require experimental investigation.

For those measuring *T*_skin_ for research, clinical, or applied purposes, points of consideration include:

- Accurate quantification of an absolute *T*_skin_ (e.g., 35.5°C) is difficult and caution needs to be applied when taking and interpreting such data, particularly when comparing *T*_skin_ across studies when the method of measurement varies.
- Equivalent measurements of *T*_skin_ cannot be assumed when different components of the sensor setup or different environments are used. Thus, for sensor setup, emphasis should be placed on consistency of sensor setup parameters, at least within a study. This consistency includes the sensor used, attachment, and (as much as practicable) the sensor-applied pressure.
- Special care needs to be taken when the temperature gradients within the measurement system are high (e.g., low environment temperature) because errors can become more influential.
- As above, reporting sensor setup variables, calibration information, and clear site details better facilitates the interpretation and external use of *T*_skin_ data.
- The information in Figure 9 may be used to assist sensor selection based on principles of minimising errors.
- In the design of future *T*_skin_ sensor systems, consideration should be given to retaining the practicality of simple affixable sensor systems while mitigating any temperature effects associated with the modification of skin coverage.

## Conclusion

Contact *T*_skin_ sensor setups and conditions used vary considerably and reporting of this information is often incomplete. The range of measurement comparisons examined here indicated that (1) the basic validity of commonly used surface *T*_skin_ sensors for accurately measuring *T*_skin_ remains unclear, and (2) the sensor type used and how it is used can meaningfully influence the measured value. Key setup variables need to be appropriately considered and consistently reported.

## Author contributions

All authors made contributions to the conception or design of this work and/or acquisition, synthesis, and interpretation of the data/information. BM drafted the manuscript with critical review from SA, CS, and RR. All authors approve the final version to be published and agree to be accountable for all aspects of the work.

### Conflict of interest statement

The authors declare that the research was conducted in the absence of any commercial or financial relationships that could be construed as a potential conflict of interest.

## Appendix

The 172 articles included within objective 2, survey of use, are given below: Adams et al., 2014, 2015; Almudehki et al., 2012; Altrichter et al., 2014; Amano et al., 2011; Ando et al., 2015; Bach et al., 2015a; Bain et al., 2011; Bain and Jay, 2011; Bandyopadhyay et al., 2011; Barr et al., 2011; Barwood et al., 2012, 2013, 2014, 2015, 2016; Best et al., 2012, 2014; Blacker et al., 2013; Bottoms and Price, 2014; Bourlai et al., 2012; Brade et al., 2014; Brown and Connolly, 2015; Bruning et al., 2013; Burdon et al., 2015; Byrne et al., 2011; Carlson et al., 2014; Caruso et al., 2015; Chou et al., 2011; Coombs et al., 2015; Corbett et al., 2015; Coso et al., 2011; Costello et al., 2015; Crabtree and Blannin, 2015; Cuddy et al., 2013, 2014, 2015; Daanen et al., 2015; Davey et al., 2013; De Pauw et al., 2011; De Sousa et al., 2014; Demachi et al., 2012a,b, 2013; Deren et al., 2012, 2014; Eijsvogels et al., 2014; Ely et al., 2011; Faulkner et al., 2015; Fernandes et al., 2014; Filho et al., 2015; Filingeri et al., 2014, 2015a,b; Flouris et al., 2015; Fonseca et al., 2015; Fortes et al., 2013; Gagnon et al., 2014a,b; Gagnon and Kenny, 2011; Gao et al., 2011, 2015, 2016; Gillis et al., 2015; Goh et al., 2011; Griggs et al., 2015; Guéritée et al., 2015a,b; Hayashi et al., 2012, 2014; Hillman et al., 2013; Hobson et al., 2013; Ihsan et al., 2013; Ito et al., 2013, 2015; James et al., 2014; Jovanović et al., 2014; Katica et al., 2011; Kenefick et al., 2011, 2014; Keramidas et al., 2013; Klute et al., 2014 Kounalakis et al., 2012; Lee et al., 2012, 2013, 2014a,b, 2015; Levels et al., 2012, 2013, 2014a,b; Limbaugh et al., 2013; Liu et al., 2013; MacRae et al., 2012, 2014; Matsuzuki et al., 2011; Mauger et al., 2014; Maughan et al., 2012; Mee et al., 2015; Mieras et al., 2014; Miyagawa et al., 2011; Moore et al., 2013; Mora-Rodriguez et al., 2011, 2012; Morris et al., 2014; Morrison et al., 2014a,b; Moyen et al., 2014a,b; Muller et al., 2011; Muñoz et al., 2012; Nassif et al., 2014; Niedermann et al., 2014; Noonan and Stachenfeld, 2012; Oda and Shirakawa, 2014; Oksa et al., 2014; Parker et al., 2013; Pearson et al., 2011; Périard et al., 2014; Pèriard et al., 2011, 2013; Périard and Racinais, 2015; Ping et al., 2011; Pryor et al., 2015; Pyke et al., 2015; Randall et al., 2015; Ravanelli et al., 2014; Richmond et al., 2015; Rintamäki et al., 2012; Roberge et al., 2012a,b; Sandsund et al., 2012; Schlader et al., 2011a,b,c; Shimazaki and Murata, 2015; Siegel et al., 2011; Sinclair and Brownsberger, 2013; Skein et al., 2012; Smith et al., 2013; Spitz et al., 2014; Stapleton et al., 2013; Sun et al., 2015; Suzuki et al., 2014; Temfemo et al., 2011; Teunissen et al., 2013, 2014; Tokizawa et al., 2014; Tsuji et al., 2012, 2014; Tumilty et al., 2011, 2014; Turner and Richards, 2015; Tyler and Sunderland, 2011a,b; Van Cutsem et al., 2015; Vanos et al., 2012; Veltmeijer et al., 2014; Wakabayashi et al., 2011, 2014; Waldron et al., 2015; Wang et al., 2013; Watkins et al., 2014; Watson et al., 2012; Wiggen et al., 2013; Wright et al., 2014; Wright Beatty et al., 2015; Xu et al., 2013; Yeung et al., 2016; Yogev et al., 2015; Zwolinska and Bogdan, 2012.
