# Supplementary material for: Skin Temperature Measurement Using Contact Thermometry: A Systematic Review of Setup Variables and Their Effects on Measured Values
Source: Front Physiol. 2018 Jan 30;9:29. doi: 10.3389/fphys.2018.00029 (PMC5797625; doi:10.3389/fphys.2018.00029)
Supplement: Supplementary file 1 [file DataSheet1.pdf]

Supplementary Material

Skin Temperature Measurement Using Contact Thermometry: A Systematic Review of Setup Variables and Their Effects on Measured Values

Braid A. MacRae, Simon Annaheim*, Christina M. Spengler, René M. Rossi

*** Correspondence:** Simon Annaheim: simon.annaheim@empa.ch

Content	Page
Supplementary Appendices 1–3	2
Supplementary Appendix 4	3
Supplementary Table 1	6
Supplementary Table 2	7
Supplementary Table 3	8
Supplementary Table 4	10
Supplementary Tables 5 & 6	16
Supplementary Figure 1	17
Supplementary Figure 2	19
Supplementary Figure 3	20
Supplementary Figure 4	22
Supplementary Figure 5	23
Supplementary Figure 6	25
Supplementary Figure 7	27
Supplementary Material references	30

Supplementary Material Appendix 1. Changes to the protocol

For the first objective on measurement comparisons, the original searches were performed with all dates considered but the inclusion criteria were modified to include in the formal analysis/synthesis only those published 1960 to present. The 1960s threshold was chosen due to commercialisation and advances in sensor technology. Further, the searches were found to be insufficiently effective in identifying 'old' (here, pre-1960s) articles. This observation was made by comparing the search results with pre-1960 articles identified via other means. We are confident that the search was effective in identifying the post-1960 articles because by manually searching the reference lists of included studies and citations of included studies, only one post-1960 study not returned in the formal search was located.

For data analysis and presentation, the data presented in forest plots (planned) were also summarised as groupings within a particular study (post-hoc decision). After producing the forest plots, we found the resultant number of comparisons displayed to be distracting from the data itself and, therefore, that figures summarising that information were better suited in the main text. The complete forest plots were retained (Supplementary Material) for reference and examination of specific comparisons of interest.

Supplementary Material Appendix 2. Extracted information

For measurement comparisons (objective 1), the following general study information was sought: if the study was performed in vivo and, if so, participant information (animal type, age, number of participants); if the study was performed using a physical model and, if so, the model composition and details; the number and type of contact sensors used for direct temperature comparisons; the number and type of attachments used for direct temperature comparisons; the sites used; whether single-site or pooled values were compared; and the number of replicates/sample size. For specific experimental comparisons, the following information was sought: temperature data from contact temperature sensors involved in a comparison of interest; surface type; notable other details about the specific comparison; environmental conditions (air temperature, relative humidity, air velocity); sensor and attachment type of the compared measurements; sensor calibration information; whether temperature was single-site or pooled values (and method of pooling if applicable); form in which data were presented; sample size.

For the survey of use (objective 2), the following information was sought: participant numbers and sex; activities performed during which T_{skin} was measured; T_{skin} sensor type and manufacturer/supplier and model information; calibration information; any details about sensor accuracy/uncertainty/precision etc; sensor attachment type and if the sensor was covered by the attachment; body sites at which T_{skin} was measured; if mean T_{skin}, mean body temperature, or other variables were calculated using T_{skin} data; and how the data was presented (e.g., absolute values, change scores; if any data was presented for individual sites).

Supplementary Material Appendix 3. Descriptions of objective 1 outcome subgroups

Several key concepts underpinning T_{skin} measurement were identified by the investigators and the outcome subgroups were defined according to these concepts:

1. Temperature disturbance of the surface underlying a surface sensor:
- temperature measurement comparisons that indicate how the surface itself is influenced by the placement of a surface sensor on top.

2. Thermal equilibrium of the surface sensor with the underlying temperature:
- temperature measurement comparisons that indicate if the surface sensors agree with measurements made 'more directly' of the surface below.

3. Influence of the attachment on surface sensors:
- comparisons of measurements taken from the same surface sensor, but with different attachments under otherwise the same measurement conditions.

4. Influence of the pressure applied by surface sensors:
- comparisons of measurements taken from the same surface sensor, but with different applied pressure under otherwise the same measurement conditions.

5. Influence of the environmental conditions on surface sensors:
- equivalent comparisons of measurements, but with different environmental conditions under otherwise the same measurement conditions.

6. Influence of the type of surface sensor:
- comparisons of temperature measurements from different surface sensors used under otherwise the same measurement conditions.

Supplementary Material Appendix 4. Notes on data inclusion and analysis for presentation

A) Mean difference and estimating standard deviation of the difference

Here, the mean of individual differences and the difference between separate comparator group means could be used interchangeably as the point estimate of measurement bias within a comparison and, therefore, we used 'mean difference' to encompass both.

Where limits of agreement (LoA) or the standard deviation of the mean difference (s_{diff}) were not available, s_{diff} was estimated from confidence intervals (CI) of the mean difference or standard deviations of the comparator group means separately (s_1 and s_2).

- For cases in which the CI of the mean difference was available, s_{diff} was estimated using:

$$\mathrm{SE} = (\text{upper CI limit} - \text{lower CI limit})/(2 \cdot t_{n-1})$$

  and

$$s_{diff} = \mathrm{SE} \cdot \sqrt{n}$$

  where SE is the standard error, t_{n-1} is the corresponding critical value from the t-distribution, and n is the sample size.

- For cases in which only s_1 and s_2 were available, s_{diff} was estimated using (Williamson et al., 2002):

$$s_{diff}^2 = s_1^2 + s_2^2 - 2r\sqrt{(s_1^2 \cdot s_2^2)}$$

where s_1 and s_2 are the standard deviations of comparators 1 and 2, respectively, and r is the correlation between the two comparators. In all cases it was a reasonable assumption that comparators had underlying measurement pairings (i.e., were not independent groups). When r was not available, it was estimated using data from a similar comparison in another study (such cases are noted below).

- For studies reporting LoA directly (using a multiplier of 2 or 1.96), we back-calculated s_{diff} and recalculated LoA using t_{n-1}. The purpose of the recalculation was for consistency in the calculation of LoA throughout the review.

B) Notes from included studies and estimates of mean difference and limits of agreement

Temperature disturbance of the surface underlying a surface sensor

- Mahanty and Roemer (1979a), mean and s_{diff} of the comparisons at each heat flux.

Thermal equilibrium of the surface sensor with the underlying temperature

- Lee et al. (1994). Human: used those with complete data from the 3 participants ('AR25, SS15, SS10, AR25, SS15, SS10'); s_{diff} for the repetitions pooled. Physical model: used the reported differences as replicates.
- Mahanty and Roemer (1979a), mean and s_{diff} of the comparisons at each heat flux.
- Psikuta et al. (2014), data given as point estimates (group means; n=3) only.

Influence of the attachment on the temperature measured by surface sensors

- Dollberg et al. (1994), data for individuals extracted from figure.
- Buono and Ulrich (1998), only s of group means given so s_{diff} estimated using correlation coefficient calculated using the 3-layer attachment data from elsewhere (Tyler, 2011).
- Deng and Liu (2008), the uncovered condition was not directly comparable so was not included. Three thermocouples were used adjacent to each other with all three covered by the attachments. We calculated a within-participant mean of the three thermocouples for each attachment, giving a point estimate (with no appropriate s for calculation of LoA). Data was extracted from figures.
- Tyler (2011), mean difference and s_{diff} reported (uncovered as reference condition).
- Psikuta et al. (2014), aluminium tape chosen as the common comparator because it was typically closest to the plate temperature. Point estimates calculated as the pooled main effect; s_{diff} estimated because only group means were available.
- Priego Quesada et al. (2015), s_{diff} estimated from the reported CI.

Influence of the pressure applied by surface sensors

- Jirak et al. (1975), mean difference and s_{diff} available; only point estimates given for rectangular sensor data.
- Mahanty and Roemer (1979b), data given as point estimates (group means; n=4) of the ~steady state, but no variance estimate was reported in the original article.

Influence of the environmental conditions on surface sensors

- Buono and Ulrich (1998), only s of group means given so s_{diff} estimated using correlation coefficient calculated using the attachment data from elsewhere (Tyler, 2011).
- van Marken Lichtenbelt et al. (2006), s_{diff} available directly for the 'grand means' only; for individual time points, s_{diff} estimated from the separate group s and using the average correlation coefficient from elsewhere (Harper Smith et al., 2010), who also used iButtons and hot and cool environments.
- Tyler (2011), mean difference and s_{diff} reported (uncovered as reference condition).
- Psikuta et al. (2014), 35°C air temperature and 0.5m/s wind velocity chosen as the common comparators because they gave the closest values to plate temperature. Point estimates calculated as the pooled main effect; s_{diff} estimated because only group means were available.
- Harper Smith et al. (2010), mean difference and s_{diff} available.

Influence of the type of surface sensor

- van Marken Lichtenbelt et al. (2006), s_{diff} available directly for the 'grand means' only; for individual time points, s_{diff} estimated from the separate group s and using the average correlation coefficient from elsewhere (Harper Smith et al., 2010), who also used iButtons and hot and cool environments.
- Harper Smith et al. (2010), mean difference and s_{diff} available.
- Bach et al. (2015), information available in the paper for the grand means for rest, exercise, and recovery; the authors had made the data available (Bach, 2014) so, in addition, we calculated mean difference and s_{diff} for the additional timings that correspond to Figure 1 in the original article.
- McFarlin et al. (2015), individual differences were presented but for all data (30 participants, 5 periods) with the pooled mean bias and LoA; pooled data used as estimates. Mean bias estimated with data extracted from figures.
- Yakovlev and Utekhin (1965), data given for maximal divergence (n=10) from which the mean difference and s_{diff} was calculated.
- Yakovlev and Utekhin (1966), only point estimates given.
- Jirak et al. (1975), only point estimates given.
- Youhui et al. (2010), only point estimates given, although reportedly 8 participants; data extracted from figure.
- James et al. (2014), mean differences and LoA reported.
- Flesch et al. (1976), point estimates only of comparisons at the temperature peaks; data extracted from figures.
- Krause (1993), values converted from °F to °C; thermocouple used as common comparator because it was typically closest to the set plate temperature. Only s of group means given so s_{diff} estimated using the mean correlation coefficient for thermistors versus thermocouple on a physical model from elsewhere (James et al., 2014).
- James et al. (2014), mean differences and LoA reported.
- Psikuta et al. (2014), PRT100 chosen as the common comparator because it was typically closest to the plate temperature. Point estimates calculated as the pooled main effect; s_{diff} estimated because only group means were available.

Supplementary Material Table 1. Database search strategies for measurement comparisons (objective 1) using Ovid Medline (**A**) and Scopus (**B**). Searches performed 13.07.2016

(A) Ovid Medline

Search step	Search terms	Results
1	exp thermometers/	3412
2	exp thermometry/	7110
3	thermomet*.ti,ab.	5500
4	(temperature* adj3 sensor*).ti,ab.	1920
5	(temperature* adj2 measur*).ti,ab.	12130
6	thermistor*.ti,ab.	1587
7	thermocouple*.ti,ab.	2304
8	(ibutton* or i-button*).ti,ab.	46
9	1 or 2 or 3 or 4 or 5 or 6 or 7 or 8	27315
10	exp uncertainty/	8276
11	uncertaint*.ti,ab.	63682
12	exp data accuracy/	242
13	exp dimensional measurement accuracy/	224
14	accurac*.ti,ab.	283440
15	validity.ti,ab.	128625
16	error*.ti,ab.	223913
17	bias*.ti,ab.	144650
18	exp calibration/	33013
19	calibrat*.ti,ab.	75762
20	correct*.ti,ab.	460863
21	exp "reproducibility of results"/	321619
22	reproducib*.ti,ab.	127897
23	repeatab*.ti,ab.	24884
24	reliab*.ti,ab.	363375
25	reference.ti,ab.	317565
26	compar*.ti,ab.	4147152
27	difference*.ti,ab.	1889488
28	agreement.ti,ab.	209855
29	exp methods/	611564
30	10 or 11 or 12 or 13 or 14 or 15 or 16 or 17 or 18 or 19 or 20 or 21 or 22 or 23 or 24 or 25 or 26 or 27 or 28 or 29	6812393
31	exp skin/	199943
32	exp temperature/	369387
33	31 and 32	5599
34	exp skin temperature/	9150
35	(skin adj2 temperature*).ti,ab.	7752
36	33 or 34 or 35	17580
37	((surface* adj2 temperature*) and tissue*).ti,ab.	430
38	((tissue* adj2 surface*) and temperature*).ti,ab.	166
39	((tissue* adj2 temperature*) and surface*).ti,ab.	242
40	37 or 38 or 39	733
41	36 or 40	18170
42	9 and 30 and 41	1558

Table continued on next page

Supplementary Material Table 1. Continued

(B) Scopus

Search step	Search terms	Results
1	TITLE-ABS-KEY(thermomet* OR (temperature W/3 sensor*) OR (temperature W/2 measur*) OR thermistor* OR thermocouple* OR ibutton* OR i-button*)	263,600
2	TITLE-ABS-KEY((uncertaint* OR accurac* OR validity OR error* OR bias* OR calibrat* OR correct* OR reproducib* OR repeatab* OR reliab* OR reference OR compar* OR difference* OR agreement*) AND temperature*)	1,355,262
3	TITLE-ABS-KEY ((skin W/2 temperature*) OR ((surface* W/2 temperature*) AND tissue*) OR ((tissue* W/2 surface*) AND temperature*) OR ((tissue* W/2 temperature*) AND surface*))	22,443
4	#1 AND #2 AND #3	2,741

Note: the influence of not including 'cutaneous temperature' was trialled for the Medline search, included in step 41 as '36 or 40 or (cutaneous adj2 temperature*).ti,ab.' This new search yielded 8 additional unique search results, none of which met the inclusion criteria. Similarly, for the Scopus search, 'TITLE-ABS-KEY (cutaneous W/2 temperature*) was included as an 'or' in step 3. This new search yielded 19 additional unique search results, none of which met the inclusion criteria. Both additional searches were done 21.07.2016

Supplementary Material Table 2. Database search strategies for the survey of use (objective 2), using Ovid Medline and performed 13.07.2016

Search step	Search terms	Results
1	exp skin temperature/an, ph [analysis, physiology]	1981
2	(skin adj3 temperatur*).ti,ab.	8335
3	1 or 2	9156
4	exp exercise/	142764
5	exp exercise test/	55222
6	exp physical exertion/	54551
7	exp sports/	147848
8	exp human engineering/	49389
9	exp walking/	24871
10	exp running/	16061
11	exp bicycling/	9037
12	physical activity.ti,ab.	72795
13	4 or 5 or 6 or 7 or 8 or 9 or 10 or 11 or 12	381062
14	3 and 13	1378
15	limit 14 to humans	1303
16	limit 15 to yr="2011 -Current"	313

Supplementary Material Table 3. Risk of bias criteria and judgements for objective 1 (measurement comparisons). The bias domains and corresponding sources of bias are adapted from the Cochrane Collaboration's risk of bias tool (Higgins et al., 2011); the criteria for judgements have been defined by the investigators specifically for this review. The sources of bias under 'other bias' have been added by the investigators for relevance to this review. The seven sources of bias evaluated in this review are numbered

**Bias domain**	**Source of bias**	**Criteria for judgement as low, unclear, or high risk of bias**
Selection bias (*systematic differences between baseline characteristics of the groups that are compared*)	1. Sequence generation	*Method used to allocate sensors/variables being compared*. Here, 'sequence' encompasses testing order (temporal allocation) and, where possible, placement of the sensors (spatial allocation).<br>Low: allocation was achieved using randomised or pseudo-randomised (for balancing) methods and stated as such, or the allocation inherently represents low risk of bias.<br>Unclear: allocation sequence/methods not reported or not clear, therefore unable to determine order.<br>High: allocation was not random or balanced (e.g., likely that one sensor type always tested before the other; left and right sides of the body used but the same sensors are always on the same side).
Selection bias	Allocation concealment	*Knowledge about the forthcoming allocations*. Allocation concealment is considered not relevant here and, therefore, will not be assessed.
Performance bias<br>(*systematic differences in exposure to factors other than the interventions of interest*)	2. Blinding of participants and personnel	*Steps taken to mitigate effects associated with knowledge of the sensor setup*. For the studies likely to be identified, concealment is expected to be unlikely to be undertaken, although possible in principle. The actual implications of blinding on temperature measurements are not clear and, therefore, a lack of blinding is considered to be 'unclear risk of bias.'<br>Low: blinding was performed adequately.<br>Unclear: blinding was partial, not performed, or not reported.<br>High: information that indicates participants or personnel were likely influenced by knowledge of the sensor type.
Detection bias	Blinding of outcome assessment	*Steps taken to mitigate effects associated with knowledge of the outcome assessors.* Blinding of outcome assessment (i.e., temperature measurement) is considered not relevant here and, therefore, will not be assessed.
Attrition bias<br>(*systematic differences between groups in withdrawals from a study*)	3. Incomplete outcome data	*Exclusion of data from particular participants/models or sensors due to withdrawal or unavailability of data from particular participants/models or sensors*.<br>Low: statement about complete data (e.g., participant completion, no missing measurements) or statement of incomplete data with suitable method reported for dealing with missing data.<br>Unclear: insufficient information to assess the likelihood of bias due to incomplete data.<br>High: information that indicates that the results were likely influenced by incomplete data.

Table continued on next page

Supplementary Material Table 3. Continued

Bias domain	Source of bias	Criteria for judgement as low, unclear, or high risk of bias
Reporting bias (*systematic differences between reported and unreported findings*)	4. Selective reporting	*Reporting of data/results selected according to particular outcomes (e.g., those that are significantly different or demonstrate a particular feature/finding) rather than being based on a priori decisions.* Includes data that was recorded and not made available. Also includes comparisons made but not reported that could bias the interpretation according to the review objective.<br>Low: the specific time points/periods reported and ways of pooling data is clearly intuitive from the methods reported, or a statement is made that the analyses were predefined and completed without changes, or all recorded data is available.<br>Unclear: insufficient information to assess the likelihood of selective analysis/presentation.<br>High: information that indicates data analysis and presentation is based on decisions made following data collection, or information that indicates particular data has been omitted without being justified.
Other bias	5. Consistency of other test conditions	*Any experimental conditions, beyond the manipulated variables of interest, which may influence one measurement differently from the corresponding comparison measurement (e.g., environmental conditions, measurement timing).*<br>Low: key conditions reported (e.g. environment, activities, timings) and tests performed in a suitably controlled fashion.<br>Unclear: insufficient information to assess whether the test conditions were suitably consistent.<br>High: information that indicates the test conditions could bias results (e.g., corresponding tests performed under different environments, measurements not taken at equivalent times).
Other bias	6. Calibration of sensors	*Baseline comparability of the sensors under the same conditions*.<br>Low: all sensors appropriately calibrated/corrected/checked by the investigators or on behalf of the investigators, or manufacturer provided a certified calibration prior to purchase.<br>Unclear: insufficient information to determine whether all the sensors were calibrated/corrected/checked.<br>High: one or more of the sensors were used according to manufacturer specifications without being certified or individually checked.
Other bias	7. Study support	*Involvement of partners that may have competing interests in the study outcome.*<br>Low: contains equivalents of both ‘conflict of interest’ and ‘acknowledgements’ sections/statements and appears to be free of support (funding or provision of equipment) from partners with potentially competing interests.<br>Unclear: insufficient information to determine study support; does not contain equivalents of both ‘conflict of interest’ and ‘acknowledgements’ sections/statements.<br>High: The study received support (funding or provision of equipment) from partners with potentially competing interests.

Supplementary Material Table 4. General information of included studies for objective 1 (measurement comparisons). Studies are categorised in accordance with the outcome comparisons (summary and forest plots) and risk of bias assessments (Supplementary Material Figure 1)

Study and surface used	Participant or model information (mean ± *s* unless otherwise specified)	Environment(s) (mean ± *s* unless otherwise specified)	Temperature sensor information †	Attachment information ‡	Sites measured	Comparison using single site or multi-site mean
1. Temperature disturbance of the surface underlying a surface sensor						
(Mahanty and Roemer, 1979a)<br><br>Physical model	Phenolic plate (thermal conductivity "similar to tissue"), surface painted black. Surface temperature range 28.7-32.5°C, in 5 steps corresponding to different heat fluxes. Model well described. Heated from beneath and insulated. 44 TC mounted at various positions; one 0.4 mm below the top surface from which surface temperature was extrapolated.	21.7 ± 0.3°C	● Thermistor probe; designed by the authors and described in detail in the paper. Applied pressure can be controlled. Thermistor (0.33 mm diameter glass bead; Veco Inc) attached to a stainless steel disk (19.1 mm diameter) that has a combination of reflective and flat black surface to mitigate influences of the probe on heat transfer from the skin.<br>● TC embedded 0.4 mm below the plate surface. Surface temperature of the plate extrapolated from the subsurface value by assuming a linear variation in temperature through the plate.	● Surface probe 'manually' held.<br>● TC embeded sub-surface	Centre top plate surface and sub-surface	NA
2. Thermal equilibrium of the surface sensor with the underlying temperature						
(Lee et al., 1994)<br><br>Human skin	n: 3<br>age (y): NR<br>mass (kg): NR<br>height (m): NR	NR	● TC (within skin); 6, 10-cm long, 29-guage TC needles (type MT25/5, Physitemp Instruments Inc.).<br>● Fibre-optic temperature sensors (Luxtron; surface of the skin); 12 used.	● TC inserted into the skin.<br>● Attachment NR for fibre-optic sensors	Upper thigh; area of ~60 mm x 60 mm. Sensors arranged in a grid, with surface sensors forming a square around the inserted skin sensors. The temperature offset for each inserted sensor was calculated separately compared to the 4 nearest surface sensors. The locally calculated offsets were then averaged together.	Single area, but mean of sites within the area
(Mahanty and Roemer, 1979a)	*See information under '1. Temperature disturbance…' above.*					
(Lee et al., 1994)<br><br>Physical model	Copper surface (0.04 cm thick sheet); 3 copper-constantan TC soldered to inner surface. Copper ~35°C. Exposed surface area 2 cm x 6 cm. Perfused water bolus (308 cm^3) beneath copper surface; water input within 1 cm of surface. Water-based coupling gel used between model and surface probes to simulate perspiration.	NR	● TC (copper-constantan)<br>● Fibre-optic temperature sensors (Luxtron® type mpm)	● 3 TC soldered to underside of copper plate.<br>● Attachment NR for fibre-optic sensors ("placed"). Water-based coupling gel was used on the upper surface and fibre-optic probes.	Copper sheet inner and outer surface	NA
(Psikuta et al., 2014)<br><br>Physical model	Aluminium plate; area 66 cm x 54 cm, thickness 1 cm. Heating elements at back surface and insulated with mineral wool. Plate temperature set at 36.5°C, controlled within ±0.03°C by PID controller and PRT100 foil sensor (Minco) within the plate. Plate outer surface painted matte black to approximate emissivity of skin.	● For wind velocity ~0.5 m/s: 16.2 ± 0.2°C, 52.4 ± 0.7%; 20.9 ± 0.1°C, 49.6 ± 0.4%; 25.7 ± 0.1°C, 48.3 ± 0.5%, 30.5°C ± 0.1°C, 48.4 ± 0.8%, 35.4°C ± 0.1°C, 48.6 ± 0.6% RH<br>● For wind velocity ~1.2 m/s: 15.7 ± 0.1°C, 51 ± 0.1%; 25.5 ± 0.1°C, 41 ± 0.8% 35.3 ± 0.1°C, 41 ± 0.4% RH	● PRT100 foil (Minaco); accuracy ±0.3°C<br>● Insulated PRT100 on a stainless steel base (Grant Instruments Ltd); accuracy ±0.3°C<br>● iButton digital thermometer (DS1922L; Maxim Integrated Products); 16 mm diameter; accuracy ±0.5°C<br>● Thermistor in silicon (MSR Electronics GmbH); accuracy ±0.2°C (note: pictures of sensors in article with mm scale)	● Aluminium tape (Tesa AG)<br>● Fixomull non-woven fabric tape (BSN medical GmbH)<br>● Tegaderm transparent film (3M Health Care)<br>● Micropore non-woven fabric tape (3M Health Care)	Plate surface and reference inside plate	NA
3. Influence of the attachment on surface sensors						
(Dollberg et al., 1994)<br><br>Human skin	n: 10<br>age (y): NR (pre-term infants)<br>mass (kg): 1.0–1.8 (range)<br>height (m): NR	Air temp measured: 32.9 ± 0.2°C in the 'tape' condition; 32.3 ± 0.3°C in the 'shield' condition. Environment was a infant incubator; skin temperature was one of the variables that influenced environmental temperature.	● Thermistor (Premie Probe No. 68-209-07; Air-Shields)	● Transpore clear tape (3M Health Care), 12 mm wide and 0.05 mm thick.<br>● Shielding spot cover (Air-Shields Critter Cover) 16 mm thick, made of polyethylene foam with aluminated polyester reflective film (Mylar) and acrylic adhesive.	1: Right back, midway between lower costal margin and top of the ilium	Single site

Study and surface used	Participant or model information (mean ± *s* unless otherwise specified)	Environment(s) (mean ± *s* unless otherwise specified)	Temperature sensor information †	Attachment information ‡	Sites measured	Comparison using single site or multi-site mean
(Buono and Ulrich, 1998)<br><br>Human skin	n: 10<br>age (y): 28 ± 5<br>mass (kg): 76 ± 3<br>height (m): NR	3 separate sessions: 23°C & 50% RH, 35°C & 80% RH, 42°C & 20% RH	● Thermistors (YSI series 400; Yellow Springs Instruments), connected to a Yellow Springs Instruments (model 47) scanning telethermometer	● Adhesive foam patch (Yellow Springs Instruments #4009 Heart Patch) and two layers of Dermiform (Johnson and Johnson Products) hypoallergenic knitted tape (25 mm wide).<br>● 'Uncovered'; acrylic ring and elastic bands. The outer surface of the sensor "freely exposed to the environment"	3: chest, dorsal surface of the forearm, lateral calf. Weightings: 0.5, 0.14, 0.36, respectively.	Multi-site mean
(Deng and Liu, 2008)<br><br>Human skin	n: 1<br>age (y): NR<br>mass (kg): NR<br>height (m): NR	~20.5°C	● TC (copper-constantan); accuracy of ±0.1°C. Three of the same type used side-by-side at the same time, exposed to each covering at the same time.	● Fabric bandage 0.5 mm thick<br>● Fabric bandage 1 mm thick<br>Fabric not described; properties used in subsequent numerical modelling but values taken from other sources.	1: proximal forearm	Single site
(Tyler, 2011)<br><br>Human skin	n: 11<br>age (y): 29 ± 4<br>mass (kg): 79 ± 15<br>height (m): 1.78 ± 0.12	Temperate conditions (23.9 ± 1.1°C; 50 ± 6% RH); warm conditions (30.1 ± 0.1°C; 43 ± 7% RH); hot conditions (35.2 ± 0.1°C; 40.1 ± 7.6% RH). Minimal air movement.	● Thermistors (THERM 37904, Viamed Ltd) connected to a digital reader (Thermistor Thermometer 5831, DigiTec Corporation)	● 'Uncovered'. 'Held' in contact with the skin for 1 min prior to data recording<br>● One layer: surgical acrylic film dressing (Tegaderm, 3M Healthcare)<br>● Two layers: surgical acrylic film dressing (Tegaderm, 3M Healthcare) and hypoallergenic surgical tape (Transpore, 3M Healthcare)<br>● Three layers: Surgical acrylic film dressing (Tegaderm, 3M Healthcare), hypoallergenic surgical tape (Transpore, 3M Healthcare) and surgical bandage (Coban, 3M Healthcare)<br>Note: sternal notch always only attached with one layer of Tagaderm tape.	4: sternal notch (same sensor used in all coverage conditions); anterior forearm, anterior thigh, calf (mid-belly and mid-length). Weightings: 0.3 sternal notch, 0.3 forearm, 0.2 thigh, 0.2 calf.	Multi-site mean
(Psikuta et al., 2014)	*See information under '2. Thermal equilibrium…' above.*					
(Priego Quesada et al., 2015)<br><br>Physical model	400 mm x 400 mm cotton fabric (213 g/m^2) on a horizontal hot plate; plate surface temperature set at 35.00 ± 0.05°C. Dry test: cotton fabric in dry state on the hot plate. Wet test: cotton fabric homogeneously wetted with 508 ± 45 g/m^2 of water using a domestic washing machine after spinning phase.	NR	● iButton (Type DS1922L, Maxim Integrated Products), accuracy of ±0.5°C; 16 mm steel can.	● Uncovered (no attachment)<br>● Hypafix clinical tape (BSN Medical GmbH)	Fabric surface	NA

Continued on next page

Study and surface used	Participant or model information (mean ± *s* unless otherwise specified)	Environment(s) (mean ± *s* unless otherwise specified)	Temperature sensor information †	Attachment information ‡	Sites measured	Comparison using single site or multi-site mean
4. Influence of the pressure applied by surface sensors						
(Yakovlev and Utekhin, 1966)<br><br>Human skin	n: NR<br>age (y): NR<br>mass (kg): NR<br>height (m): NR	NR	● Nickel wire (diameter 70 µm) wound in a zig-zag grid "in a single plane".<br>● Thermistor, horizontally arranged (within the same plane as the mounting).<br>● Thermistor, perpendicular to the skin surface when mounted.<br>● Type NR, perpendicular to the skin surface when mounted.<br>● Thermistor, mounted in Plexiglas disc/ring.<br>● Thermistor, 400 mm^2 area of contact with the skin.<br>● Thermistor, mounted in the apex of a cone (perpendicular to the skin surface).<br>(note: sensors drawn in original article)	● NC; at least one with "cement no. 88"	NR	NR
(Guadagni et al., 1972)<br><br>Human skin	n: NR<br>age (y): NR<br>mass (kg): NR<br>height (m): NR	NR; environment "kept constant"; care taken to understand stability of skin temperature	● Thermistor, glass-coated (0.32 mm diamter) housed in a specially made probe for hand-held use; tip diameter 2.5 mm. Designed to have minimal infuence on heat transfer from the skin and is described in considerable detail in the paper.	● 'Manually' held	NR	NC
(Jirak et al., 1975)<br><br>Human skin	n: 14<br>age (y): NR<br>mass (kg): NR<br>height (m): NR	26 ± 1°C; "minimal" air movement	● Thermistor thermometer probe with a circular contact, diameter 3.7 mm, area 10.8 mm^2<br>● Thermistor thermometer with a rectangular contact of 4.2 mm x 6.5 mm, area 27.3 mm^2<br>The probes were fixed to the arm of a microbal-ance (note that the authors use the mass in g and refer to the mass itself as 'pressure').	● Both probes 'manually' held	5: forehead, forearm, dorsal hand, stomach, tibia	Single site
(Mahanty and Roemer, 1979b)<br><br>Human skin	n: 4<br>age (y): 28 ± 6<br>mass (kg): NR<br>height (m): 1.8	21.6 ± 0.4°C	● Thermistor probe; designed by the authors and described in detail in the paper. The applied pressure can be controlled using weights. Thermistor (0.34 mm glass coated bead; Veco, Inc) mounted in the underside of a aluminium disc (0.46 mm, 19 mm diameter) painted black and mounted to a phenolic supporting rod.	● 'Manually' held.	2: mid-ventral thigh, lateral greater trochan-ter	Single site
5. Influence of the environmental conditions on surface sensors						
(Buono and Ulrich, 1998)	*See information under '3. Influence of the attachment…' above.*					
(van Marken Lichtenbelt et al., 2006)<br><br>Human skin	n: 6<br>age (y): 23 ± 2<br>mass (kg): 67 ± 4<br>height (m): NR	34.9 ± 0.1 °C, 8.6 ± 0.5% RH, and 15.5 ± 0.3°C and 64.0 ± 2.3% RH	● TC (ULTRAKUST Electronic GmbH).<br>● iButton (DS1291H; Maxim Integrated) semi-conductor temperature sensor; 16 mm diameter, 6 mm height. Manufacture specifications: tem-perature range +15 to +46 °C, accuracy of 1 °C, precision of 0.125 °C.	● Leukoplast tape (BSN medical GmbH)	14 (ISO 9886, 2000). Mean temperature calculated according to 14 sites (equally weighted), or from only 4 of those sites (neck, 0.28; scapula, 0.28; hand, 0.16; shin, 0.28)	Multi-site mean
(Harper Smith et al., 2010)<br><br>Human skin	n: 8<br>age (y): 27 ± 5<br>mass (kg): 82 ± 7<br>height (m): 1.80 ± 0.06<br>body fat (%): 13.3 ± 6.0	10, 20, and 30°C sessions. In each session, rest at low wind velocity (0.18 m/s) and higher wind velocity (2.30 m/s); cycle ergometry at higher wind velocity (2.30 m/s). 40% RH through-out. A 30-min period at a dry bulb tem-perature of 25°C separated each seg-ment.	● Thermistor (EUS-U, Grant Instruments Ltd); resolution 0.01°C<br>● iButton (Maxim Integrated); 16 mm (diameter) 6 mm height; resolution 0.0625°C	● Hypafix tape (BSN Medical GmbH)	14 during rest, 8 during exercise (ISO 9886, 2004).	Multi-site mean
(Tyler, 2011)	*See information under '3. Influence of the attachment…' above.*					

Continued on next page

Study and surface used	Participant or model information (mean ± *s* unless otherwise specified)	Environment(s) (mean ± *s* unless otherwise specified)	Temperature sensor information †	Attachment information ‡	Sites measured	Comparison using single site or multi-site mean
(Yakovlev and Utekhin, 1966)<br><br>Physical model	Limited detail. Suede surface; metal reservoir filled with water (~33°C).	NC; example given with environmental temperature change from 47°C to 23°C over 70 min but in text a constant 20°C gradient noted	● Nickel wire (diameter 70 μm) wound in a zig-zag grid "in a single plane".<br>● Thermistor, horizontally arranged (within the same plane as the mounting).<br>● Thermistor, perpendicular to the skin surface when mounted.<br>● Type NR, perpendicular to the skin surface when mounted.<br>● Thermistor, mounted in Plexiglas disc/ring.<br>● Thermistor, 400 mm^2 area of contact with the skin.<br>● Thermistor, mounted in the apex of a cone (perpendicular to the skin surface).<br>(note: sensors drawn in original article)	● NC; at least one with "cement no. 88"	Suede surface	NA
(Psikuta et al., 2014)	*See information under '2. Thermal equilibrium…' above.*					
6. Influence of the type of surface sensor						
(van Marken Lichtenbelt et al., 2006)	*See information under '5. Influence of the environmental conditions…' above.*					
(Harper Smith et al., 2010)	*See information under '5. Influence of the environmental conditions…' above.*					
(Bach et al., 2015)<br><br>Human skin	n: 30<br>age (y): 25 ± 3<br>mass (kg): 79 ± 11<br>height (m): 1.82 ± 0.08<br>BMI (kg m^{-2}): 23.9 ± 2.2<br>sum of 8 skin folds (mm): 88.9 ± 32.3	30 min rest (24.0 ± 1.2°C, 56 ± 8%, <0.1 m s-1 air speed), 30 min cycle in the heat (38.0 ± 0.5°C, 41 ± 2%, 0.5 ± 0.1 m s-1 air speed), and 45 min recovery (24.0 ± 1.3°C, 56 ± 9%, <0.1 m s-1 air speed).	● Thermistor (EU-UU-VL5–0, Grant Instruments) connected to data logger (Grant Instruments); sensitivity 0.01°C, uncertainty ±0.1°C<br>● iButton (DS1922L-F50; Maxim Intergrated); sensitivity 0.0625°C, uncertainty ±0.5°C	● Single layer of white Leuko Sportstape (Beiersdorf)	4: neck, scapula, hand, shin (weightings: 0.28, 0.28, 0.16, 0.28, respectively)	Multi-site mean
(McFarlin et al., 2015)<br><br>Human skin	n: 30<br>age (y): 22 ± 4<br>mass (kg): NR<br>height (m): NR<br>BMI 23.4 ± 1.8<br>body fat (%) 18.1± 3.5	39.2 ± 0.2°C; 55 ± 5% RH; "static" air	● Thermistors ('wired'; YSI400 series; Yellow Springs Instruments) connected to a tele-thermometer (Cole-Parmer). Manufacturer-stated accuracy of 0.1ºC.<br>● iButtons (DS1921H; Maxim Integrated). Manufacturer-stated accuracy of 0.125ºC.	● Cover-Roll adhesive tape (BSN Medical)	2: bicep, abdomen	Single site
(Yakovlev and Utekhin, 1965)<br><br>Human skin	n: NC<br>age (y): 20–24<br>mass (kg): NR<br>height (m): NR	20 ± 0.5°C in control room, 45 ± 1°C in chamber.	● Nickel wire (diameter 70 μm) wound in a zig-zag grid "in a single plane". "Between the wire loops of the sensor is a space (0.5 mm) through which sweat freely passes and evaporates." Sensor height "equal to the diameter of the nickel wire"; area 15mm x 40 mm.<br>● Type NR; "point" sensor (diameter 0.3-1 mm)<br>● Type NR; plane sensor (area 15 mm x 25 mm)	● Attachment NR	Forehead	Single site
(Yakovlev and Utekhin, 1966)<br><br>Human skin	n: NR<br>age (y): NR<br>mass (kg): NR<br>height (m): NR	NR	● Nickel wire (diameter 70 μm) wound in a zig-zag grid "in a single plane".<br>● Thermistor, horizontally arranged (within the same plane as the mounting).<br>● Thermistor, perpendicular to the skin surface when mounted.<br>● Type NR, perpendicular to the skin surface when mounted.<br>● Thermistor, mounted in Plexiglas disc/ring.<br>● Thermistor, 400 mm^2 area of contact with the skin.<br>● Thermistor, mounted in the apex of a cone (perpendicular to the skin surface).<br>(note: sensors drawn in original article)	● NC; at least one with "cement no. 88"	Forearm, "lower third of the inner surface"	Single site

Study and surface used	Participant or model information (mean ± *s* unless otherwise specified)	Environment(s) (mean ± *s* unless otherwise specified)	Temperature sensor information †	Attachment information ‡	Sites measured	Comparison using single site or multi-site mean
(Jirak et al., 1975)<br><br>Human skin	n: NC<br>age (y): NR<br>mass (kg): NR<br>height (m): NR	26 ± 1°C; "minimal" air movement	● Thermistor thermometer probe with a circular contact, diameter 3.7 mm, area 10.8 mm^2<br>● Thermistor thermometer with a rectangular contact of 4.2 mm x 6.5 mm, area 27.3 mm^2<br>Note: the probes were fixed to the arm of a microbalance; the pressure is confounded due to same mass but different area between probe types.	● Both probes 'manually' held	5: forehead, forearm, dorsal hand, stomach, tibia	Single site
(Mahanty and Roemer, 1979a)<br><br>Human skin	n: 1<br>age (y): NR<br>mass (kg): NR<br>height (m): NR	NR	● Thermistor probe; designed by the authors and described in detail in the paper. Thermistor (0.33 diameter glass bead; Veco Inc) attached to a stainless steel disk (19.1 mm diameter) that has a combination of reflective and flat black surface to mitigate influences of the probe on heat transfer from the skin.<br>● TC; no other information	● Thermistor probe 'manually' held.<br>● TC "taped"; no other information.	Mid-ventral thigh; hair removed locally	Single site
(Mahanty and Roemer, 1979b)<br><br>Human skin	n: NR<br>age (y): NR<br>mass (kg): NR<br>height (m): NR	21.6 ± 0.4°C	● Thermistor probe; designed by the authors and described in detail in the paper. The applied pressure can be controlled using weights. Thermistor (0.34 mm glass coated bead; Veco, Inc) mounted in the underside of a aluminium disc (0.46 mm, 19 mm diameter) painted black and mounted to a phenolic supporting rod.<br>● TC; no other information	● Thermistor probe 'manually' held.<br>● TC "taped"; no other information.	2: mid-ventral thigh, lateral greater trochanter	NC
(Youhui et al., 2010)<br><br>Human skin	n: 8<br>age (y): NR<br>mass (kg): NR<br>height (m): NR	NR	● PRT1000 film (2.08 x 1.32 x 0.36 mm^3) welded onto a printed circuit board (12 x 2.5 x 0.2 mm^3) and covered with an "insulating compound" painted on the surface to protect from sweat.<br>● "Standard thermometer"; no other information reported	● NC; likely 'surgical tape'	4: right upper arm, left chest, right thigh, right leg. Compared as single sites and as mean skin temperature (weightings: 0.3, 0.3, 0.2, 0.2, respectively)	Single site and multi-site mean
(James et al., 2014)<br><br>Human skin	n: 14<br>age (y): 38 ± 11<br>mass (kg): 77 ± 7<br>height (m): 1.79 ± 0.08<br>sum of skinfolds (mm): 33.6 ± 7.7	31.9 ± 1°C, 61 ± 9% RH	● Thermistors ('wired'; EUS-U-VS5-0, Eltek Ltd.) connected to a data logger (GrantSquirrel 1000 series, Grant Instruments Ltd.). Manufacturer stated accuracy was ±0.2 °C.<br>● Thermistors (telemetry system; four skin thermistors, ELEU-U-VS-02, Eltek Ltd.) connected to a transmitter (Gen II GD38, Eltek Ltd.). Data transmitted wirelessly to a data logger (RX250AL 1000 series Wireless Logger, Eltek Ltd.). Manufacturer stated accuracy was ±0.1 °C.	● Tegaderm "breathable film patches" (3M Healthcare)	4: mid-belly of the pectoralis major, biceps brachii, rectus femoris and gastrocnemius on the right of the body. Pooled with weightings: 0.3 chest, 0.3 arm, 0.2 thigh, 0.2 calf	Multi-site mean
(Yakovlev and Utekhin, 1965)<br><br>Physical model	A metal reservoir filled with warm water (~35-37°C). Layer of polyvinyl chloride (0.5 mm) and layer of chamois glued on the outside surface of the reservoir	NR	● Nickel wire (diameter 70 µm) wound in a zig-zag grid "in a single plane". "Between the wire loops of the sensor is a space (0.5 mm) through which sweat freely passes and evaporates." Sensor height "equal to the diameter of the nickel wire"; area 15 mm x 40 mm.<br>● Type NR; "point" sensor (diameter 0.3-1 mm)	● Attachment NR	Chamois surface	NA

Continued on next page

Study and surface used	Participant or model information<br>(mean ± *s* unless otherwise specified)	Environment(s)<br>(mean ± *s* unless otherwise specified)	Temperature sensor information †	Attachment information ‡	Sites measured	Comparison using single site or multi-site mean
(Flesch et al., 1976)<br><br>Physical model	Excised (24 h after death) 1 mm thick, human epidermis on a simulated hypodermis (sand or rock salt), with tube(s) parallel below surface to create hot spots. Model kept at a constant temperature by a waterbath (37°C). Depth under surface, diameter, and temperature of the tube(s) able to be changed. Model is well described in paper.	NR; studies done when "room temperature is constant"	● Thermistor (Valvo, 0.35 mm in diameter)<br>● TC (Philips, 0.25 mm in diameter)<br>Both sensor types integrated in a "precision thermometer probe".	● 'Manually' held	Model (skin) surface	NA
(Krause, 1993)<br><br>Physical model	Steel plate (area 102 mm x 102 mm; Applied Thermol, Inc) with flat surface; temperature controlled by two heat-sensing elements with "inherent accuracy of ± 1.3°F" (0.7°C); plate settings over range 27–38°C.	~27°C, ~60% RH	● Thermistor (STS-400; Respiratory Supprt Products Inc)." Temperature sensing component 2 x 9 mm"; thermistor "sandwiched between a plastic film and foam covered with a metallic layer". Accuracy reported as ±0.2°C in the range of 0 to 60°C. Monitor used: RSP Model TM-20'D (SN 2398D) and RSP monitor cable (catalog C400-10).<br>● Thermistor (SHER-I-TEMP; Sheridan Catheter Corp) "Temperature sensing component contact 2 x 8 mm." Thermistor embedded in insulation foam, adhesive layer, and a reflective metallic layer. Accuracy reported as ±0.1°C in the range of 0 to 60°C. Monitor used: Sheridan Sonatemp 400/700 temperature monitor.<br>● TC (Mon-a-therm, Mallinckrodt Inc). "Temperature sensing component contact 16 mm x 16 mm." Accuracy reported as ±0.1°C in the range of 1 to 50°C	● All sensors self-adhesive (have an adhesive bottom surface)	Plate surface	NA
(James et al., 2014)<br><br>Physical model; i) corrected and ii) uncorrected thermistors	Cast iron block (150 x 120 x 120 mm^3) placed in a temperature-controlled waterbath (Fischer Scientific DMU19), submerged to within 1 mm of the surface. Water bath temperature 33, 35, and 38°C.	NR	● TC (Type K probe attached to Fluke 51 II instrument, range 200 °C to 1000 °C, divisions 0.1 °C)<br>● Thermistors ('wired'; EUS-U-VS5-0, Eltek Ltd.) connected to a data logger (GrantSquirrel 1000 series, Grant Instruments Ltd.). Manufacturer stated accuracy was ±0.2 °C.<br>● Thermistors (telemetry system; four skin thermistors, ELEU-U-VS-02, Eltek Ltd.) connected to a transmitter (Gen II GD38, Eltek Ltd.). Data transmitted wirelessly to a data logger (RX250AL 1000 series Wireless Logger, Eltek Ltd.). Manufacturer stated accuracy was ±0.1 °C.<br>Two subsets used in this review: thermistors prior to correction and (the same) thermistors following correction.	● Tegaderm "breathable film patches" (3M Healthcare)	Centre top plate surface	NA
(Psikuta et al., 2014)	*See information under '2. Thermal equilibrium…' above.*					

n, number of participants; NA, not applicable; NC, not clear; NR, not reported; PRT, platinum resistance thermometer; RH, relative humidity; s, standard deviation; TC, thermocouples

† Sensor information was summarised here as reported in the original paper for usage of terms like accuracy. Accuracy is a qualitative term (and should, therefore, not be given a numerical value), whereas uncertainty and resolution are quantitative and can be given a numerical value

‡ The phrase 'manually held' represents the sensor being held by an entity that remains independent of the surface (e.g., an experimenter). This contrasts with other fixation methods (e.g., tape, other adhesives)

Supplementary Material Table 5. Number of sites used during the measurement of T_{skin} in studies involving physical activity (objective 2—survey of use)

Number of sites	NR	1	2	3	4	5	6	7	8	9	10	11	12	13	14	15	16
Count (studies)	4	13	5	10	79	7	9	11	18	4	6	1	2	1	1	0	1
Percentage	2.3	7.6	2.9	5.8	45.9	4.1	5.2	6.4	10.5	2.3	3.5	0.6	1.2	0.6	0.6	0.0	0.6

NR, not reported

Supplementary Material Table 6. Body sites used during the measurement of T_{skin} in studies involving physical activity (objective 2—survey of use)

Body site	Number of studies including site (%)	Number of studies including more than one sensor at site (%)
Face/forehead	47 (27.3)	5 (2.9)
Neck	16 (9.3)	3 (1.7)
Shoulder	7 (4.1)	0 (0.0)
Chest and sternum	127 (73.8)	0 (0.0)
Abdomen	21 (12.2)	1 (0.6)
Scapula or upper back	38 (22.1)	0 (0.0)
Lower back or para-vertebral	9 (5.2)	1 (0.6)
Non-specific 'back'	15 (8.7)	0 (0.0)
Arm (upper arm/ biceps/ triceps)	96 (55.8)	0 (0.0)
Forearm	56 (32.6)	0 (0.0)
Hand	41 (23.8)	0 (0.0)
Finger	11 (6.4)	1 (0.6)
Anterior thigh[a]	122 (70.9)	1 (0.6)
Posterior thigh	11 (6.4)	0 (0.0)
Anterior leg (shin)	16 (9.3)	0 (0.0)
Posterior leg (calf)[b]	105 (61.0)	0 (0.0)
Ankle	1 (0.6)	1 (0.6)
Foot (incl. toes)	18 (10.5)	3 (1.7)
Other	4 (2.3)	2 (1.2)
Specific sites not reported in text	10 (5.8)	–

a If non-specific ‘thigh’ was reported, it was assumed to be anterior thigh
b If non-specific ‘leg’ was reported, it was assumed to be posterior leg (calf)

Supplementary Material Figure 1. Risk of bias summary for individual subsets included in the measurement comparisons, listed in accordance with the arrangement of the summary and forest plots

Study	Year	In vivo / model	Data in forest plots?	Sequence generation	Blinding of participants and personnel	Incomplete outcome data	Selective reporting	Other: Consistency of test conditions	Other: Calibration/baseline comparability of sensors	Other: Study support
**1. Temperature disturbance of the surface underlying a surface sensor**										
Mahanty and Roemer	1979a	Model	Y	?	?	?	+	+	?	?
**2. Thermal equilibrium of the surface sensor with the underlying temperature**										
Lee et al.	1994	In vivo	Y	+	?	+	+	?	+	?
Mahanty and Roemer	1979a	Model	Y	?	?	?	+	+	?	?
Lee et al.	1994	Model	Y	+	?	?	?	?	?	?
Psikuta et al.	2014	Model	Y	?	?	?	+	+	+	?
**3. Influence of the attachment on surface sensors**										
Dollberg et al.	1994	In vivo	Y	–	?	?	?	–	?	?
Buono and Ulrich	1998	In vivo	Y	?	?	?	?	+	+	?
Deng and Liu	2008	In vivo	Y	?	?	?	?	?	+	?
Tyler	2011	In vivo	Y	+	?	?	?	+	+	?
Psikuta et al.	2014	Model	Y	?	?	?	+	+	+	?
Priego Quesada et al.	2015	Model	Y	–	?	?	+	?	?	+
**4. Influence of the pressure applied by surface sensors**										
Yakovlev and Utekhin	1966	In vivo	N	?	?	?	?	?	?	?
Guadagni et al.	1972	In vivo	N	?	?	?	?	+	+	?
Jirak et al.	1975	In vivo	Y	?	?	?	?	?	+	?
Mahanty and Roemer	1979b	In vivo	Y	?	?	?	+	+	+	?
**5. Influence of the environmental conditions on surface sensors**										
Buono and Ulrich	1998	In vivo	Y	?	?	?	?	?	+	?
van Marken Lichtenbelt et al.	2006	In vivo	Y	?	?	?	?	?	+	+
Harper Smith et al.	2010	In vivo	Y	?	?	?	?	?	+	?
Tyler	2011	In vivo	Y	?	?	?	?	?	+	?
Yakovlev and Utekhin	1966	Model	N	?	?	?	?	?	?	?
Psikuta et al.	2014	Model	Y	?	?	?	+	+	+	?

- + Low risk of bias
- ? Unclear risk of bias
- – High risk of bias

Figure continued on next page

Supplementary Material Figure 1. Continued

Study	Year	In vivo / model	Data in forest plots?	Sequence generation	Blinding of participants and personnel	Incomplete outcome data	Selective reporting	Other: Consistency of test conditions	Other: Calibration/baseline comparability of sensors	Other: Study support
**6. Influence of the type of surface sensor**										
van Marken Lichtenbelt et al.	2006	In vivo	Y	?	?	?	?	?	+	+
Harper Smith et al.	2010	In vivo	Y	?	?	?	?	+	+	?
Bach et al.	2015	In vivo	Y	+	?	?	+	+	+	+
McFarlin et al.	2015	In vivo	Y	?	?	?	+	+	–	+
Yakovlev and Utekhin	1965	In vivo	Y	?	?	?	–	+	?	?
Yakovlev and Utekhin	1966	In vivo	Y	?	?	?	?	?	?	?
Jirak et al.	1975	In vivo	Y	?	?	?	?	–	+	?
Mahanty and Roemer	1979a	In vivo	N	?	?	?	?	?	?	?
Mahanty and Roemer	1979b	In vivo	N	?	?	?	–	+	+	?
Youhui et al.	2010	In vivo	Y	?	?	?	?	?	?	?
James et al.	2014	In vivo	Y	?	?	?	?	+	+	?
Yakovlev and Utekhin	1965	Model	N	?	?	?	–	+	?	?
Flesch et al.	1976	Model	Y	?	?	?	?	+	–	?
Krause	1993	Model	Y	?	?	+	+	?	–	–
James et al.	2014	Model (uncorrected)	Y	?	?	?	?	?	–	?
James et al.	2014	Model (corrected)	Y	?	?	?	?	?	+	?
Psikuta et al.	2014	Model	Y	?	?	?	+	+	+	?

+ Low risk of bias   ? Unclear risk of bias   – High risk of bias

Supplementary Material Figure 2. Temperature disturbance of the surface underlying a surface sensor (mean difference between comparators and 95% limits of agreement)

Study and features	Comparator 1	Comparator 2	Mean difference [95% LoA], °C (negative = comparator 2 < 1)	Mean difference [95% LoA], °C
**Physical model**				
Mahanty and Roemer (1979a); 22°C environment				
Phenolic plate	TC embedded in plate, surface ‘undisturbed[a]	TC embedded in plate, surface ‘disturbed[b]		-0.03 [-0.12, 0.06]
Phenolic plate	TC embedded in plate, surface ‘undisturbed[a]	Thermistor probe on surface[c]		-0.02 [-0.26, 0.22]

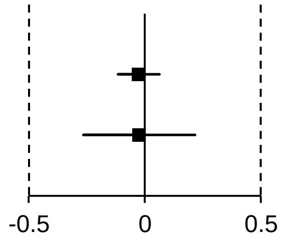

LoA, limits of agreement; TC, thermocouple

a Thermocouple 0.4 mm below the plate surface; temperature at the surface calculated by assuming linear variation in temperature through the plate

b Temperature as in ‘a’, but while a surface temperature probe is in contact with the surface

c Glass bead thermistor attached to a stainless steel disk (diameter of disk 19.1 mm); probe was designed specifically to have little influence on heat transfer between surface and environment

Supplementary Material Figure 3. Thermal equilibrium of the surface sensor with the underlying temperature (**A** and **B**). The data of Psikuta et al. (2014) are presented separately in (**B)** because presentation the number of combinations was not practical to display in the conventional format

(A)

Study and features	Comparator 1	Comparator 2	Mean difference [95% LoA], °C (negative = comparator 2 < 1)	Mean difference [95% LoA], °C
**Human skin in vivo**				
Lee et al. (1994); attachment for FO sensor NR; env NR				
Rest; upper thigh	TC needle in skin	FO surface sensor		-0.20 [-0.54, 0.14]
**Physical model**				
Mahanty and Roemer (1979a); surface sensor manually held; env NR				
Phenolic plate	TC subsurface, disturbed	Surface thermistor		0.00 [-0.16, 0.17]
Lee et al. (1994); attachment for FO sensor NR; env NR				
Copper sheet	TC soldered inner surface	FO surface probes		-0.16 [-0.99, 0.67]
Psikuta et al. (2014)				
Aluminium plate; see (B) below				

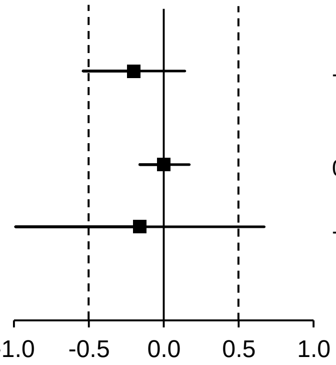

(B)

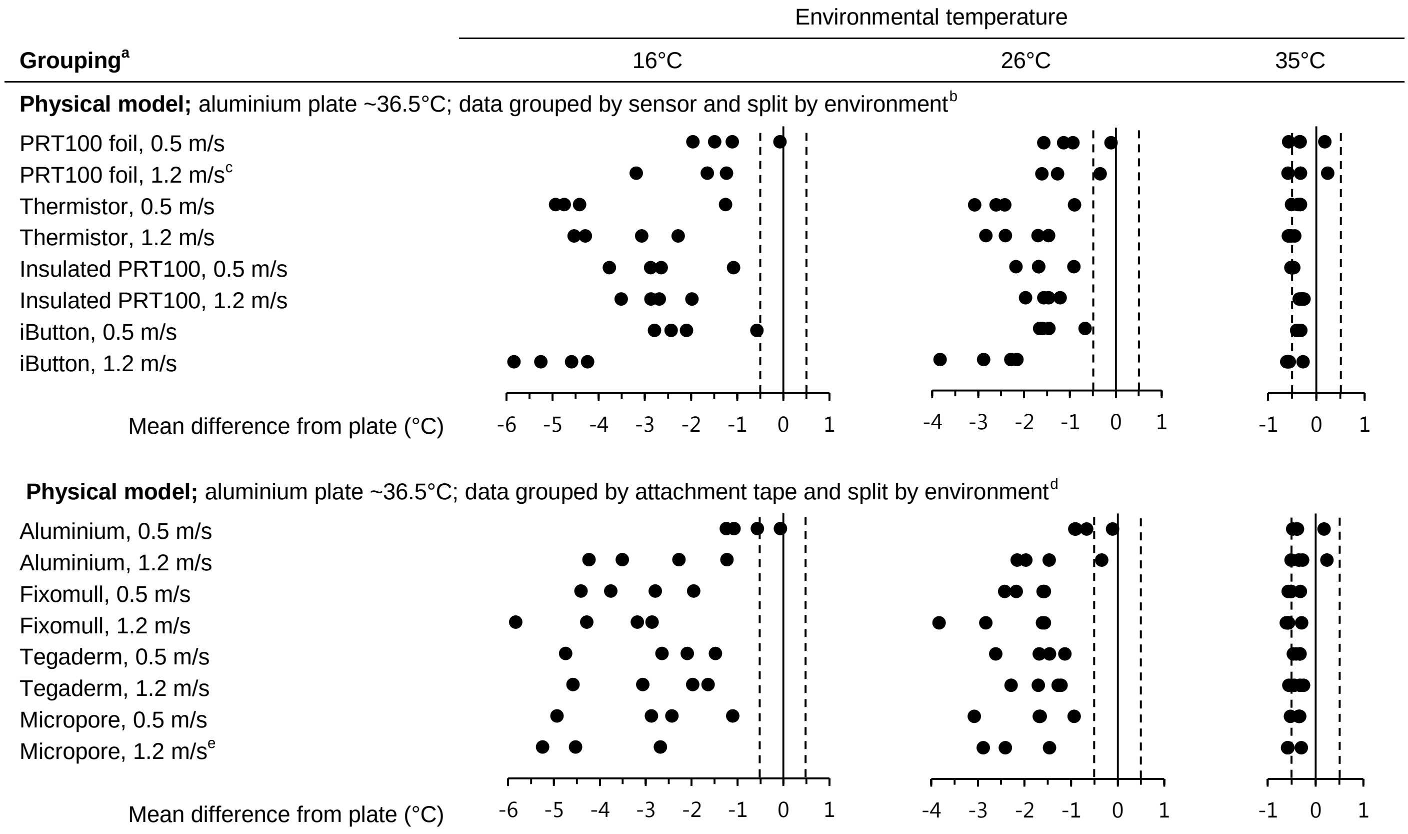

env, environment; FO, fibre optic; LoA, limits of agreement; NA, not applicable; PRT, platinum resistance thermometer; TC, thermocouple

a Data are point estimates for sensor temperature difference from plate temperature (negative indicates surface sensor lower than plate) under environmental temperatures of 16, 26, and 35°C, each with 0.5 and 1.2 m/s air velocity. Data is also available at 21 and 31°C for 0.5 m/s but is not included and only point estimates are given here, each for practicality and clarity of presentation. Note that the groupings by sensor (three top panels) and groupings by attachment (three bottom panels) represent the equivalent data, shown separately to highlight both sensor type (top) and attachment (bottom).

b The four data points within each row are attachment types (aluminium, Fixomull, Tegaderm, and Micropore tapes; order depends on data)

c Missing one tape type (Micropore)

d The four data points within each row are sensor types (PRT100 foil, thermistor, insulated PRT100, and iButton; order depends on data)

e Missing one sensor type (PRT100 foil)

Supplementary Material Figure 4. Influence of the attachment on the temperature measured by surface sensors (mean difference between comparators and 95% limits of agreement)

Study and features	Comparator 1	Comparator 2	Mean difference [95% LoA], °C
**Human skin in vivo**			
Dollberg et al. (1994); lower back; 32–33°C env			
Rest; thermistor	Transpore tape	Shielding attachment	0.35 [-0.17, 0.87]
Buono and Ulrich (1998); 3-site mean			
Ex; 23°C env; thermistor	Uncov (acrylic ring)	Foam & Demiform tape	1.30 [0.28, 2.32]
Ex; 35°C env; thermistor	Uncov (acrylic ring)	Foam & Demiform tape	0.80 [0.17, 1.43]
Ex; 42°C env; thermistor	Uncov (acrylic ring)	Foam & Demiform tape	0.40 [-0.39, 1.19]
Deng and Liu (2008); distal forearm; 21°C env			
Rest; thermocouple	Bandage, 0.5 mm	Bandage, 1.0 mm	0.50
Tyler (2011); 4-site mean[a]			
Ex, 24°C env; thermistor	Uncov (held 1 min)	1-L tape	0.06 [-0.03, 0.15]
	Uncov (held 1 min)	2-L tape	0.41 [-0.3, 1.12]
	Uncov (held 1 min)	2-L tape + 1-L bandage	0.79 [0.21, 1.37]
Ex; 30°C env; thermistor	Uncov (held 1 min)	1-L tape	0.20 [-0.47, 0.87]
	Uncov (held 1 min)	2-L tape	0.47 [-0.36, 1.30]
	Uncov (held 1 min)	2-L tape + 1-L bandage	0.76 [0.02, 1.50]
Ex; 35°C env; thermistor	Uncov (held 1 min)	1-L tape	0.53 [-0.34, 1.4]
	Uncov (held 1 min)	2-L tape	0.67 [-0.11, 1.45]
	Uncov (held 1 min)	2-L tape + 1-L bandage	0.87 [-0.11, 1.85]
**Physical model**			
Psikuta et al. (2014); Al plate painted black; pooled env[b]			
Pooled sensor type[b]	Al tape	Tegaderm tape	-1.11 [-1.80, -0.42]
	Al tape	Micropore tape	-1.17 [-2.68, 0.33]
	Al tape	Fixomull tape	-1.37 [-2.14, -0.60]
Priego Quesada et al. (2015); Al plate with cotton fabric surface; env NR			
Dry fabric; iButton	Uncov (no attach)	Hypafix tape	0.51 [0.21, 0.81]
Wetted fabric; iButton	Uncov (no attach)	Hypafix tape	0.25 [-0.35, 0.85]

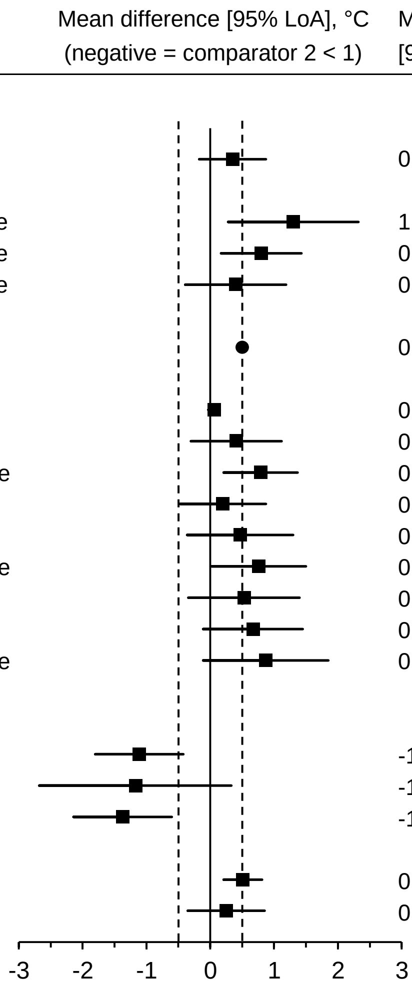

Al, aluminium; attach, attachment; env, environment; ex, exercise; L, layer; LoA, limits of agreement; PRT, platinum resistance thermometer; uncov, uncovered. Black circles used for data without LoA.

a One site (sternal notch) was always attached with 1-L Tegaderm tape irrespective of attachment of the other three sites

b Data from all sensors (PRT100 foil, thermistor, insulated PRT100, and iButton) and environments (15–35°C, 0.5 m/s) are pooled here

Supplementary Material Figure 5. Influence of the pressure applied by surface sensors (mean difference between comparators and 95% limits of agreement)

Study and features	Comparator 1 (pressure [mmHg])	Comparator 2 (pressure [mmHg])	Mean difference [95% LoA], °C
**Human skin in vivo**			
Yakovlev and Utekhin (1966); site(s), attachment NC; env NR			
Rest; 7 sensor types	Unable to display[a]		NA
Guadagni et al. (1972); site(s) and env NR			
Rest; thermistor probe	Unable to display[a]		NA
Jirak et al. (1975); sensor manually held; 26°C env[b]			
Rest; forehead; thermistor[c]	Circular (136)	Circular (34)	-0.49 [-0.99, 0.01]
	Circular (136)	Circular (68)	-0.31 [-0.7, 0.08]
	Circular (136)	Circular (272)	0.36 [-0.24, 0.96]
	Circular (136)	Circular (409)	0.73 [-0.03, 1.49]
	Circular (136)	Circular (545)	0.98 [0.27, 1.69]
	Circular (136)	Circular (681)	1.11 [0.48, 1.74]
Rest; forearm; thermistor[c]	Circular (136)	Circular (34)	-0.22 [-0.57, 0.13]
	Circular (136)	Circular (68)	-0.21 [-0.49, 0.07]
	Circular (136)	Circular (272)	0.16 [-0.1, 0.42]
	Circular (136)	Circular (409)	0.41 [-0.07, 0.89]
	Circular (136)	Circular (545)	0.62 [0.25, 0.99]
	Circular (136)	Circular (681)	0.81 [0.08, 1.54]
Rest; dorsal hand; thermistor[c]	Circular (136)	Circular (34)	-0.27 [-0.64, 0.10]
	Circular (136)	Circular (68)	-0.18 [-0.33, -0.03]
	Circular (136)	Circular (272)	0.21 [-0.09, 0.51]
	Circular (136)	Circular (409)	0.39 [0.02, 0.76]
	Circular (136)	Circular (545)	0.61 [0.18, 1.04]
	Circular (136)	Circular (681)	0.72 [0.07, 1.37]
Rest; stomach; thermistor[c]	Circular (136)	Circular (34)	-0.65 [-1.15, -0.15]
	Circular (136)	Circular (68)	-0.37 [-0.85, 0.11]
	Circular (136)	Circular (272)	0.45 [0.02, 0.88]
	Circular (136)	Circular (409)	0.89 [0.31, 1.47]
	Circular (136)	Circular (545)	1.10 [0.54, 1.66]
	Circular (136)	Circular (681)	1.33 [0.47, 2.19]
Rest; tibia; thermistor[c]	Circular (136)	Circular (34)	-0.12 [-0.47, 0.23]
	Circular (136)	Circular (68)	-0.04 [-0.28, 0.2]
	Circular (136)	Circular (272)	0.14 [-0.34, 0.62]
	Circular (136)	Circular (409)	0.25 [-0.42, 0.92]
	Circular (136)	Circular (545)	0.37 [-0.32, 1.06]
	Circular (136)	Circular (681)	0.44 [-0.27, 1.15]
Rest; forehead; thermistor[d]	Rectangular (54)	Rectangular (27)	-0.14
Rest; forearm; thermistor[d]	Rectangular (54)	Rectangular (27)	0.00
Rest; hand; thermistor[d]	Rectangular (54)	Rectangular (27)	-0.15
Rest; stomach; thermistor[d]	Rectangular (54)	Rectangular (27)	-0.28
Rest; tibia; thermistor[d]	Rectangular (54)	Rectangular (27)	-0.10
Mahanty and Roemer (1979b); sensor manually held; 22°C env			
Rest; thigh; thermistor[e]	Circular (2)	Circular (5)	0.09
Rest; thigh; thermistor[e]	Circular (2)	Circular (10)	0.31
Rest; thigh; thermistor[e]	Circular (2)	Circular (15)	0.51
Rest; thigh; thermistor[e]	Circular (2)	Circular (20)	0.60
Rest; trochanter; thermistor[e]	Circular (2)	Circular (5)	0.06
Rest; trochanter; thermistor[e]	Circular (2)	Circular (10)	0.10
Rest; trochanter; thermistor[e]	Circular (2)	Circular (15)	0.30
Rest; trochanter; thermistor[e]	Circular (2)	Circular (20)	0.30

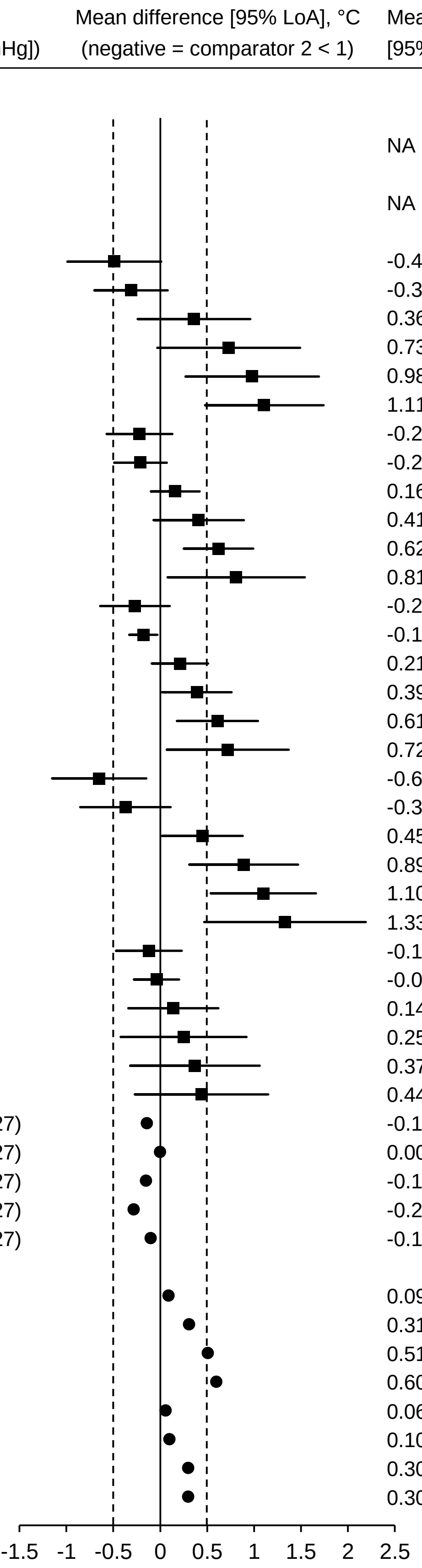

env, environment; LoA, limits of agreement; NA, not applicable; NC, not clear; NR, not reported. Black circles used for data without LoA

a Not presented here due to limited detail in the original article; see text for information

b Pressures here calculated from the surface area and reported mass

c Circular probe, diameter 3.7 mm, area 10.8 mm^2

d Rectangular probe, surface 4.2 mm x 6.5 mm, area 27.3 mm^2

e Circular probe, diameter 19 mm, area 284 mm^2

Supplementary Material Figure 6. Influence of the environmental conditions on surface sensors (mean difference between comparators and 95% limits of agreement)

Study and features	Comparator 1	Comparator 2	Mean difference [95% LoA], °C (negative = comparator 2 < 1)	Mean difference [95% LoA], °C
**Human skin in vivo**				
Buono and Ulrich (1998); 3-site mean				
23°C env; Ex; thermist	Uncov (acrylic ring)	Foam & Demiform tape		1.3 [0.28, 2.32]
35°C env; Ex; thermist	Uncov (acrylic ring)	Foam & Demiform tape		0.8 [0.17, 1.43]
42°C env; Ex; thermist	Uncov (acrylic ring)	Foam & Demiform tape		0.4 [-0.39, 1.19]
van Marken Lichtenbelt et al. (2006); 14-site mean; Leukoplast tape				
16°C env; Rest, GM	Thermocouple	iButton		0.88 [-0.69, 2.45]
35°C env; Rest, GM	Thermocouple	iButton		-0.24 [-0.63, 0.15]
16°C env; Rest, 0 min	Thermocouple	iButton		0.0 [-0.84, 0.84]
35°C env; Rest, 0 min	Thermocouple	iButton		0.3 [-0.57, 1.17]
16°C env; Rest, 7 min	Thermocouple	iButton		1.3 [0.66, 1.94]
35°C env; Rest, 7 min	Thermocouple	iButton		-0.3 [-0.48, -0.12]
16°C env; Rest, 14 min	Thermocouple	iButton		0.7 [0.03, 1.37]
35°C env; Rest, 14 min	Thermocouple	iButton		-0.2 [-0.43, 0.03]
16°C env; Rest, 22 min	Thermocouple	iButton		0.6 [0.39, 0.81]
35°C env; Rest, 22 min	Thermocouple	iButton		-0.2 [-0.38, -0.02]
16°C env; Rest, 29 min	Thermocouple	iButton		0.6 [0.34, 0.86]
35°C env; Rest, 29 min	Thermocouple	iButton		-0.1 [-0.41, 0.21]
Harper Smith et al. (2010); 12-site mean rest, 8-site mean ex; Hypafix tape				
10°C, 0.2 m/s env; rest	Thermist	iButton		1.11 [0.91, 1.31]
20°C, 0.2 m/s env; rest	Thermist	iButton		0.70 [0.65, 0.75]
30°C, 0.2 m/s env; rest	Thermist	iButton		0.28 [0.21, 0.35]
10°C, 2.3 m/s env; rest	Thermist	iButton		1.36 [1.25, 1.47]
20°C, 2.3 m/s env; rest	Thermist	iButton		0.79 [0.71, 0.87]
30°C, 2.3 m/s env; rest	Thermist	iButton		0.26 [0.21, 0.31]
10°C, 2.3 m/s env; Ex	Thermist	iButton		0.99 [0.32, 1.66]
20°C, 2.3 m/s env; Ex	Thermist	iButton		0.42 [0.26, 0.58]
30°C, 2.3 m/s env; Ex	Thermist	iButton		0.32 [0.07, 0.57]
Tyler (2011); 4-site mean[a]				
24°C env; Ex; thermist	Uncov (held 1 min)	1-L tape		0.06 [-0.03, 0.15]
30°C env; Ex; thermist	Uncov (held 1 min)	1-L tape		0.20 [-0.47, 0.87]
35°C env; Ex; thermist	Uncov (held 1 min)	1-L tape		0.53 [-0.34, 1.40]
24°C env; Ex; thermist	Uncov (held 1 min)	2-L tape		0.41 [-0.30, 1.12]
30°C env; Ex; thermist	Uncov (held 1 min)	2-L tape		0.47 [-0.36, 1.30]
35°C env; Ex; thermist	Uncov (held 1 min)	2-L tape		0.67 [-0.11, 1.45]
24°C env; Ex; thermist	Uncov (held 1 min)	1-L tape + 1-L band		0.79 [0.21, 1.37]
30°C env; Ex; thermist	Uncov (held 1 min)	2-L tape + 1-L band		0.76 [0.02, 1.50]
35°C env; Ex; thermist	Uncov (held 1 min)	2-L tape + 1-L band		0.87 [-0.11, 1.85]
**Physical model**				
Yakovlev and Utekhin (1966); suede surface; 7 sensors, attachment and env NC				
Air temperature	Unable to display[b]			NA
Psikuta et al. (2014); Al plate ~36.5°C; pooled sensors and attachments[c]				
Air temperature	35°C, 0.5 m/s env	31°C, 0.5 m/s env		-0.63 [-0.84, -0.41]
	35°C, 0.5 m/s env	26°C, 0.5 m/s env		-1.15 [-1.97, -0.33]
	35°C, 0.5 m/s env	21°C, 0.5 m/s env		-1.64 [-3.02, -0.26]
	35°C, 0.5 m/s env	16°C, 0.5 m/s env		-2.00 [-3.25, -0.75]
Air velocity	0.5 m/s, 35°C env	1.2 m/s, 35°C env		0.00 [-0.13, 0.13]
	0.5 m/s, 26°C env	1.2 m/s, 26°C env		-0.42 [-1.32, 0.48]
	0.5 m/s, 16°C env	1.2 m/s, 16°C env		-0.99 [-1.94, -0.04]

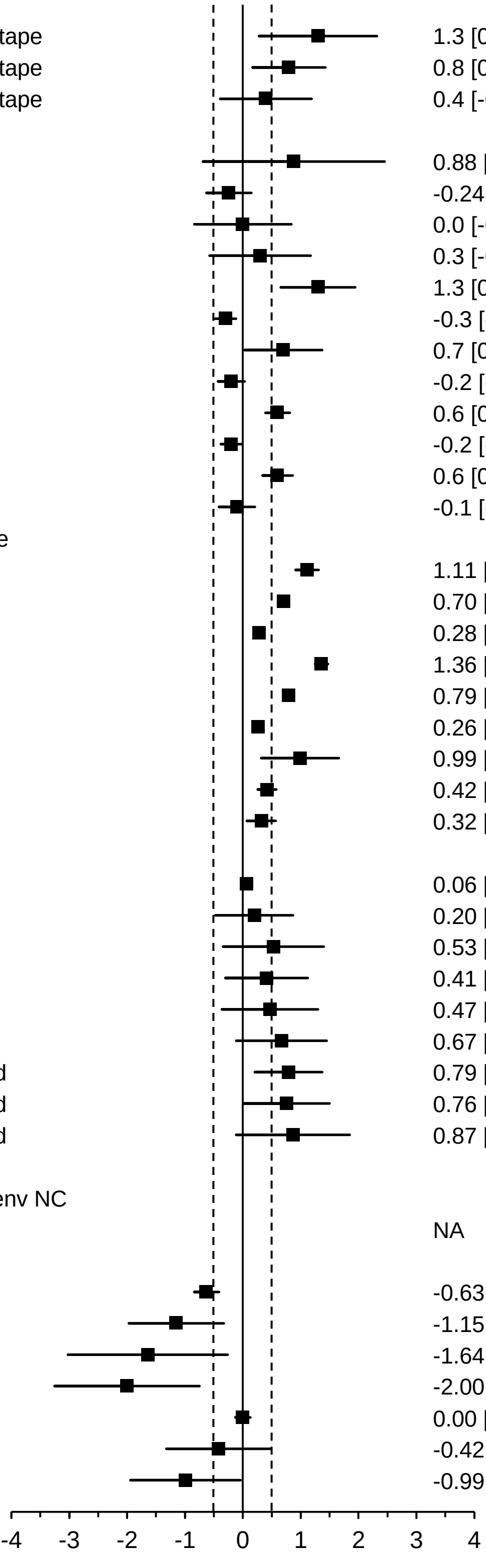

Al, aluminium; band, bandage; env, environment; ex, exercise; GM, grand mean; L, layer; LoA, limits of agreement; NA, not applicable; NC, not clear; PRT, platinum resistance thermometer; thermist, thermistor; uncov, uncovered

a One site (sternal notch) was always attached with 1-L Tegaderm tape irrespective of attachment of the other three sites

b Not presented here due to limited detail in the original article; see text for information

c Data from all sensors (PRT100 foil, thermistor, insulated PRT100, and iButton) and attachment types (aluminium, Fix-omull, Tegaderm, and Micropore tapes) are pooled here

Supplementary Material Figure 7. Influence of the type of surface sensor (mean difference between comparators and 95% limits of agreement). Data are split into three figures (**A–C**) for practicality of display

(A)

**Study and features**	Comparator 1	Comparator 2	Mean difference [95% LoA], °C
**Human skin in vivo**			
van Marken Lichtenbelt et al. (2006); 14-site mean; Leukoplast tape			
Rest, grand mean; 35°C env	Thermocouple	iButton	-0.24 [-0.63, 0.15]
Rest, 0 min; 35°C env	Thermocouple	iButton	0.3 [-0.57, 1.17]
Rest, 7 min; 35°C env	Thermocouple	iButton	-0.3 [-0.48, -0.12]
Rest, 14 min; 35°C env	Thermocouple	iButton	-0.2 [-0.43, 0.03]
Rest, 22 min; 35°C env	Thermocouple	iButton	-0.2 [-0.38, -0.02]
Rest, 29 min; 35°C env	Thermocouple	iButton	-0.1 [-0.41, 0.21]
Rest, grand mean; 16°C env	Thermocouple	iButton	0.88 [-0.69, 2.45]
Rest, 0 min; 16°C env	Thermocouple	iButton	0.0 [-0.85, 0.85]
Rest, 7 min; 16°C env	Thermocouple	iButton	1.3 [0.66, 1.94]
Rest, 14 min; 16°C env	Thermocouple	iButton	0.7 [0.03, 1.37]
Rest, 22 min; 16°C env	Thermocouple	iButton	0.6 [0.39, 0.81]
Rest, 29 min; 16°C env	Thermocouple	iButton	0.6 [0.34, 0.86]
Harper Smith et al. (2010); 12-site mean rest, 8-site mean ex; Hypafix tape			
Rest; 10°C, 0.2 m/s env	Thermistor	iButton	1.11 [0.91, 1.31]
Rest; 10°C, 2.3 m/s env	Thermistor	iButton	1.36 [1.25, 1.47]
Ex; 10°C, 2.3 m/s env	Thermistor	iButton	0.99 [0.32, 1.66]
Rest; 20°C, 0.2 m/s env	Thermistor	iButton	0.70 [0.65, 0.75]
Rest; 20°C, 2.3 m/s env	Thermistor	iButton	0.79 [0.71, 0.87]
Ex; 20°C, 2.3 m/s env	Thermistor	iButton	0.42 [0.26, 0.58]
Rest; 30°C, 0.2 m/s env	Thermistor	iButton	0.28 [0.21, 0.35]
Rest; 30°C, 2.3 m/s env	Thermistor	iButton	0.26 [0.21, 0.31]
Ex; 30°C, 2.3 m/s env	Thermistor	iButton	0.32 [0.07, 0.57]
Bach et al. (2015a); 4-site mean; Leuko tape			
Rest, grand mean; 24°C env	Thermistor	iButton	0.01 [-0.41, 0.43]
Rest, 0 min; 24°C env	Thermistor	iButton	0.04 [-0.31, 0.39]
Rest, 15 min; 24°C env	Thermistor	iButton	0.00 [-0.41, 0.41]
Rest, 30 min; 24°C env	Thermistor	iButton	0.02 [-0.41, 0.45]
Ex, grand mean; 38°C env	Thermistor	iButton	0.26 [-0.63, 1.15]
Ex, 0 min; 38°C env	Thermistor	iButton	-0.56 [-1.09, -0.03]
Ex, 6 min; 38°C env	Thermistor	iButton	0.03 [-0.34, 0.40]
Ex, 12 min; 38°C env	Thermistor	iButton	0.27 [-0.14, 0.68]
Ex, 18 min; 38°C env	Thermistor	iButton	0.47 [0.00, 0.94]
Ex, 24 min; 38°C env	Thermistor	iButton	0.57 [0.04, 1.10]
Ex, 30 min; 38°C env	Thermistor	iButton	0.57 [0.00, 1.14]
Recov rest, grand mean; 24°C	Thermistor	iButton	0.37 [-0.66, 1.4]
Recov rest, 0 min; 24°C env	Thermistor	iButton	1.05 [0.27, 1.83]
Recov rest, 6 min; 24°C env	Thermistor	iButton	0.79 [-0.07, 1.65]
Recov rest, 12 min; 24°C env	Thermistor	iButton	0.57 [-0.27, 1.41]
Recov rest, 18 min; 24°C env	Thermistor	iButton	0.39 [-0.37, 1.15]
Recov rest, 24 min; 24°C env	Thermistor	iButton	0.23 [-0.45, 0.91]
Recov rest, 30 min; 24°C env	Thermistor	iButton	0.10 [-0.58, 0.78]
Recov rest, 36 min; 24°C env	Thermistor	iButton	-0.01 [-0.71, 0.69]
Recov rest, 42 min; 24°C env	Thermistor	iButton	-0.09 [-0.79, 0.61]
McFarlin et al. (2015); Cover-Roll tape			
Ex; abdomen; 39°C env	Thermistor	iButton	-0.99 [-2.59, 0.61]
Ex; bicep; 39°C env	Thermistor	iButton	-0.69 [-2.68, 1.30]

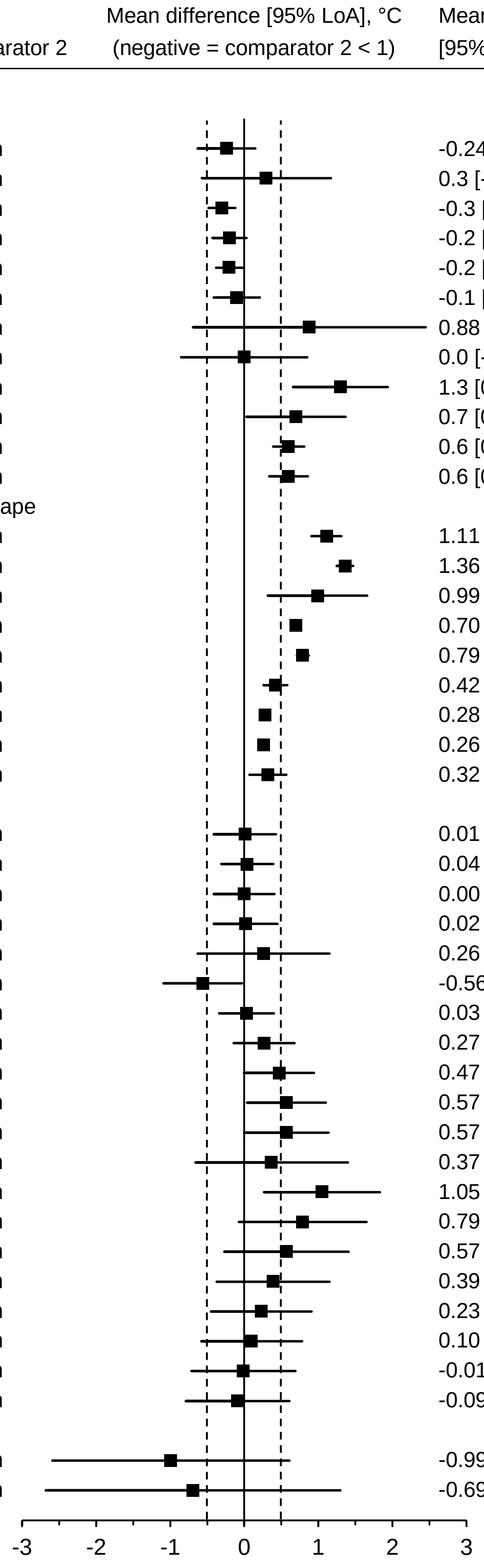

Supplementary Material Figure 7. Continued

(B)

**Study and features**	Comparator 1	Comparator 2	Mean difference [95% LoA], °C
**Human skin in vivo**			
Yakovlev and Utekhin (1965); forehead; attachment NR; 20–45°C env			
Rest & ex; mean of max diff	Nickel wire (grid)	‘Plane’ sensor	2.45 [0.85, 4.05]
Rest & ex; mean of max diff	Nickel wire (grid)	‘Point’ sensor	2.18 [-0.01, 4.37]
Yakovlev and Utekhin (1966); forearm; attachment NC; env NR			
Rest; wetted skin	Nickel wire (grid)	Thermist, horizontal	0.3
Rest; wetted skin	Nickel wire (grid)	Thermist, perpendic	1.0
Rest; wetted skin	Nickel wire (grid)	NR, perpendic	1.0
Rest; wetted skin	Nickel wire (grid)	Thermist, disc	2.0
Rest; wetted skin	Nickel wire (grid)	Thermist, plane	1.5
Rest; wetted skin	Nickel wire (grid)	Thermist, cone	2.5
Jirak et al. (1975); sensor manually held; 26°C env; custom thermist probe			
Rest; forehead	Circular[a]; 68 mmHg	Rectang[b]; 27 mmHg	1.88
Rest; forearm	Circular[a]; 68 mmHg	Rectang[b]; 27 mmHg	1.33
Rest; hand	Circular[a]; 68 mmHg	Rectang[b]; 27 mmHg	1.91
Rest; stomach	Circular[a]; 68 mmHg	Rectang[b]; 27 mmHg	0.83
Rest; tibia	Circular[a]; 68 mmHg	Rectang[b]; 27 mmHg	1.12
Rest; forehead	Circular[a]; 136 mmHg	Rectang[b]; 54 mmHg	1.66
Rest; forearm	Circular[a]; 136 mmHg	Rectang[b]; 54 mmHg	1.13
Rest; hand	Circular[a]; 136 mmHg	Rectang[b]; 54 mmHg	1.97
Rest; stomach	Circular[a]; 136 mmHg	Rectang[b]; 54 mmHg	0.80
Rest; tibia	Circular[a]; 136 mmHg	Rectang[b]; 54 mmHg	1.13
Mahanty and Roemer (1979a); mid-thigh; env NR			
Thermist probe vs TC taped to skin; unable to display[c]			NA
Mahanty and Roemer (1979b); mid-thigh and trochanter; 22°C env			
Thermist probe vs TC taped to skin; unable to display[c]			NA
Youhui et al. (2010); tape; env NR			
Rest; leg	‘Standard thermom’	PRT1000	0.24
Rest; chest	‘Standard thermom’	PRT1000	-0.17
Rest; thigh	‘Standard thermom’	PRT1000	0.05
Rest; upper arm	‘Standard thermom’	PRT1000	0.12
Rest; 4-site mean	‘Standard thermom’	PRT1000	0.04
James et al. (2014); 4-site mean; Tegaderm tape; 32°C env			
Rest, 10 min	Thermist (wired)	Thermist (telemetry)	-0.09 [-0.53, 0.35]
Rest, 20 min	Thermist (wired)	Thermist (telemetry)	-0.16 [-0.65, 0.33]
Rest, 25 min	Thermist (wired)	Thermist (telemetry)	-0.13 [-0.54, 0.28]
Rest, 30 min	Thermist (wired)	Thermist (telemetry)	-0.15 [-0.56, 0.26]
Rest, 35 min	Thermist (wired)	Thermist (telemetry)	-0.12 [-0.54, 0.30]
Ex, 4 min	Thermist (wired)	Thermist (telemetry)	-0.23 [-0.55, 0.09]
Ex, 8 min	Thermist (wired)	Thermist (telemetry)	-0.25 [-0.48, -0.02]
Ex, 12 min	Thermist (wired)	Thermist (telemetry)	-0.22 [-0.60, 0.16]
Ex, 16 min	Thermist (wired)	Thermist (telemetry)	-0.24 [-0.74, 0.26]
Ex, 20 min	Thermist (wired)	Thermist (telemetry)	-0.24 [-0.88, 0.40]

Mean difference [95% LoA], °C (negative = comparator 2 < 1)

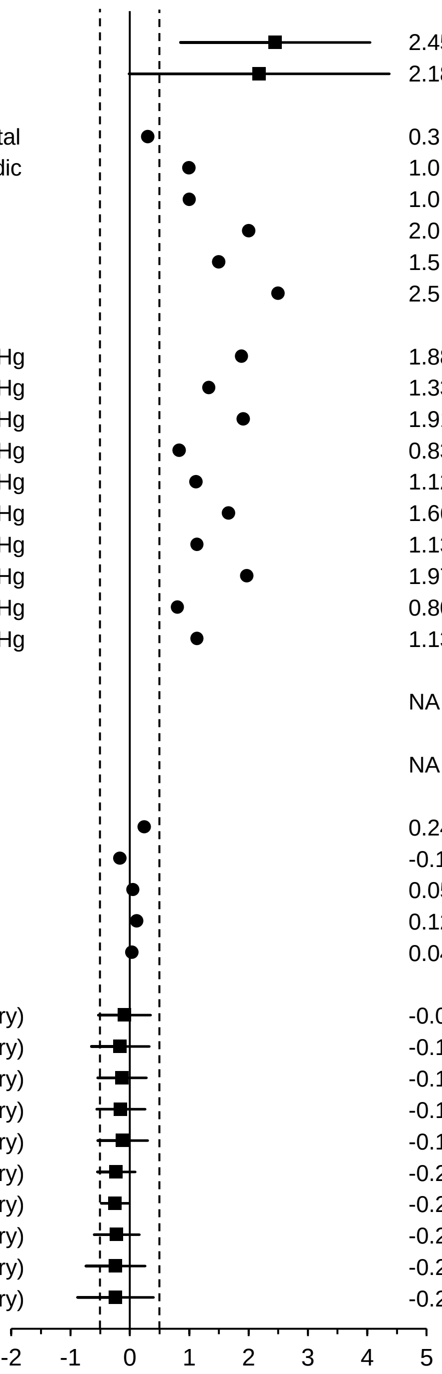

Supplementary Material Figure 7. Continued

(C)

**Study and features**	Comparator 1	Comparator 2	Mean difference [95% LoA], °C (negative = comparator 2 < 1)	Mean difference [95% LoA], °C
**Physical model**				
Yakovlev and Utekhin (1965); chamois surface; attachment and env NR				
Nickel wire (grid) vs. 'point' sensor; unable to display[c]				NA
Flesch et al. (1976); excised human skin; sensor manually held; env NR				
Single temperature peak	Thermist probe	TC probe		-0.40
Double temperature peak	Thermist probe	TC probe		-0.05
Krause (1993); steel plate; self-adhesive sensors; 27°C env				
Plate ~29°C	TC disc	Thermist 1		-0.42 [-1.09, 0.25]
Plate ~29°C	TC disc	Thermist 2		-0.23 [-0.56, 0.10]
Plate ~32°C	TC disc	Thermist 1		-1.02 [-1.87, -0.17]
Plate ~32°C	TC disc	Thermist 2		-1.30 [-1.84, -0.76]
Plate ~35°C	TC disc	Thermist 1		-0.98 [-1.93, -0.03]
Plate ~35°C	TC disc	Thermist 2		-1.36 [-2.80, 0.08]
Plate ~38°C	TC disc	Thermist 1		-1.26 [-2.03, -0.49]
Plate ~38°C	TC disc	Thermist 2		-2.21 [-3.34, -1.08]
James et al. (2014); iron block; Tegaderm tape; env NR				
Thermist uncorrected	TC (type K)	Thermist (wired)		-0.22 [-0.43, -0.01]
Thermist uncorrected	TC (type K)	Thermist (telemetry)		0.15 [-0.10, 0.40]
Thermist corrected	TC (type K)	Thermist (wired)		0.08 [-0.25, 0.41]
Thermist corrected	TC (type K)	Thermist (telemetry)		0.02 [-0.22, 0.26]
Psikuta et al. (2014); Al plate; pooled attachments and env[d]				
Plate ~36.5°C	PRT100 foil	iButton		-0.42 [-0.89, 0.05]
Plate ~36.5°C	PRT100 foil	Insulated PRT100		-0.81 [-1.37, -0.25]
Plate ~36.5°C	PRT100 foil	Thermist		-1.35 [-2.17, -0.53]

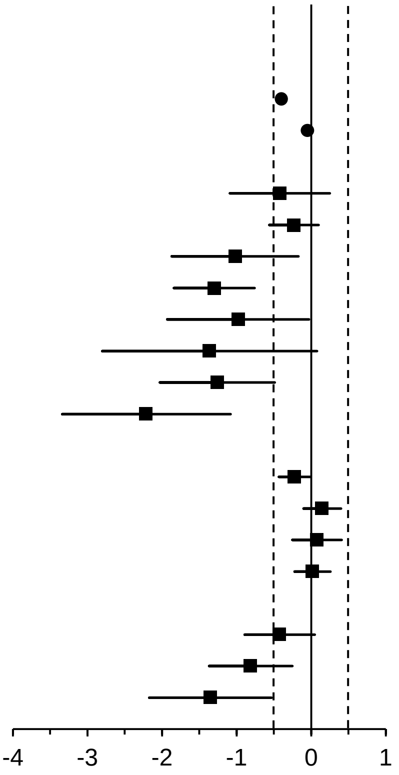

Al, aluminium; diff, difference; env, environment; ex, exercise; LoA, limits of agreement; max, maximum; NA, not applicable; NC, not clear; NR, not reported; perpendic, perpendicular; PRT, platinum resistance thermometer; recov, recovery; rectang, rectangular; TC, thermocouple; thermist, thermistor; thermom, thermometer. Black circles used for data without LoA

a Circular probe, diameter 3.7 mm, area 10.8 mm^2

b Rectangular probe, surface 4.2 mm x 6.5 mm, area 27.3 mm^2

c Not presented here due to the limited information reported in original article; see text for information

d Data from all attachment types (aluminium, Fixomull, Tegaderm, and Micropore tapes) and environments (15–35°C, 0.5 m/s) are pooled here

References for supplementary information

Bach, A. (2014). A comparison between conductive and infrared devices for measuring mean skin temperature at rest, during exercise in the heat, and recovery: figshare. Available at: https://doi.org/10.6084/m9.figshare.1254250.v1 [Accessed December 8, 2016].

Bach, A. J. E., Stewart, I. B., Disher, A. E., and Costello, J. T. (2015). A comparison between conductive and infrared devices for measuring mean skin temperature at rest, during exercise in the heat, and recovery. *PLoS One* 10, e0117907. doi:10.1371/journal.pone.0117907.

Buono, M. J., and Ulrich, R. L. (1998). Comparison of mean skin temperature using "covered" versus "uncovered" contact thermistors. *Physiol. Meas.* 19, 297–300.

Deng, Z.-S., and Liu, J. (2008). Effect of fixing material on skin-contact temperature measurement by wearable sensor. in *Proc. 5th Int. Workshop on Wearable and Implantable Body Sensor Networks, BSN2008, in conjunction with the 5th Int. Summer School and Symp. on Medical Devices and Biosensors, ISSS-MDBS 2008* (IEEE), 137–140. doi:10.1109/ISSMDBS.2008.4575037.

Dollberg, S., Atherton, H. D., Sigda, M., Acree, C. M., and Hoath, S. B. (1994). Effect of insulated skin probes to increase skin-to-environmental temperature gradients of preterm infants cared for in convective incubators. *J. Pediatr.* 124, 799–801.

Flesch, U., Wegener, O. H., Scheffler, A., and Ernst, H. (1976). Thermometry of the surface of human skin. A study on a model using thermocouples, thermistors, thermovision and thermodyes. *Phys. Med. Biol.* 21, 422–428.

Guadagni, D. N., Kreith, F., Smyth, C. J., and Bartholomew, B. A. (1972). Contact probe for skin temperature measurements. *J. Phys. E.* 5, 869–876. doi:10.1088/0022-3735/5/9/012.

Harper Smith, A. D., Crabtree, D. R., Bilzon, J. L. J., and Walsh, N. P. (2010). The validity of wireless iButtons(R) and thermistors for human skin temperature measurement. *Physiol. Meas.* 31, 95–114.

Higgins, J. P. T., Altman, D. G., and Sterne, J. A. C. (2011). "Chapter 8: Assessing risk of bias in included studies," in *Cochrane Handbook for Systematic Reviews of Interventions*, eds. J. P. T. Higgins and S. Green (The Cochrane Collaboration).

James, C. A., Richardson, A. J., Watt, P. W., and Maxwell, N. S. (2014). Reliability and validity of skin temperature measurement by telemetry thermistors and a thermal camera during exercise in the heat. *J. Therm. Biol.* 45, 141–149. doi:10.1016/j.jtherbio.2014.08.010.

Jirak, Z., Jokl, M., Stverak, J., Pechlat, R., and Coufalov, H. (1975). Correction factors in skin temperature measurement. *J. Appl. Physiol.* 38, 752–756.

Krause, B. F. (1993). Accuracy and response time comparisons of four skin temperature-monitoring devices. *Nurse Anesth.* 4, 55–61.

Lee, E. R., Kapp, D. S., Lohrbach, A. W., and Sokol, J. L. (1994). Influence of water bolus temperature on measured skin surface and intradermal temperatures. *Int. J. Hyperth.* 10, 59–72.

Mahanty, S. D., and Roemer, R. B. (1979a). Skin temperature probe. *J. Biomech. Eng.* 101, 232–238.

Mahanty, S. D., and Roemer, R. B. (1979b). The effect of pressure on skin temperature measurements for a disk sensor. *J. Biomech. Eng.* 101, 261–266.

McFarlin, B. K., Venable, A. S., Williams, R. R., and Jackson, A. W. (2015). Comparison of techniques for the measurement of skin temperature during exercise in a hot, humid environment. *Biol. Sport* 32, 11–14.

Priego Quesada, J. I., Martínez Guillamón, N., de Anda, R. M. C. O., Psikuta, A., Annaheim, S., Rossi, R. M., et al. (2015). Effect of perspiration on skin temperature measurements by infrared thermography and contact thermometry during aerobic cycling. *Infrared Phys. Technol.* 72, 68–76. doi:10.1016/j.infrared.2015.07.008.

Psikuta, A., Niedermann, R., and Rossi, R. M. (2014). Effect of ambient temperature and attachment method on surface temperature measurements. *Int. J. Biometeorol.* 58, 877–885.

Tyler, C. J. (2011). The effect of skin thermistor fixation method on weighted mean skin temperature. *Physiol. Meas.* 32, 1541–1547.

van Marken Lichtenbelt, W. D., Daanen, H. A. M., Wouters, L., Fronczek, R., Raymann, R. J. E. M., Severens, N. M. W., et al. (2006). Evaluation of wireless determination of skin temperature using iButtons. *Physiol. Behav.* 88, 489–497.

Williamson, P. R., Lancaster, G. A., Craig, J. V, and Smyth, R. L. (2002). Meta-analysis of method comparison studies. *Stat. Med.* 21, 2013–2025. doi:10.1002/sim.1158.

Yakovlev, V. V, and Utekhin, B. A. (1965). Errors in skin temperature measurements due to changes in evaporation under the sensor. *Bull. Exp. Biol. Med.* 60, 1210–1212. doi:10.1007/BF00793268.

Yakovlev, V. V, and Utekhin, B. A. (1966). Criteria for the evaluation of thermoelectric transducers and tests of certain designs of such transducers for measurements of skin temperature. 227–231.

Youhui, P., Zhiwei, L., and Li, P. (2010). Skin temperature measurement method. *J. Southeast Univ. (English Ed.* 26, 258–261.
